# Design, Synthesis and Anti-Platelet Aggregation Activity Study of Ginkgolide-1,2,3-triazole Derivatives

**DOI:** 10.3390/molecules24112156

**Published:** 2019-06-07

**Authors:** Jian Cui, Le’An Hu, Wei Shi, Guozhen Cui, Xumu Zhang, Qing-Wen Zhang

**Affiliations:** 1 State Key Laboratory of Quality Research in Chinese Medicine, Institute of Chinese Medical Sciences, University of Macau, Macau 999078, China; mb65822@umac.mo (J.C.); yb67526@umac.mo (L.H.); 2 Department of Chemistry and Shenzhen Grubbs Institute, Southern University of Science and Technology, Shenzhen 518000, China; 3 Department of Bioengineering, Zhuhai Campus of Zunyi Medical University, Zhuhai 519041, Guangdong, China; diana1354726@outlook.com

**Keywords:** ginkgolide, platelet-activating factor receptor, inhibitor

## Abstract

Ginkgolides are the major active component of *Ginkgo biloba* for inhibition of platelet activating factor receptor. An azide-alkyne Huisgen cycloaddition reaction was used to introduce a triazole nucleus into the target ginkgolide molecules. A series of ginkgolide-1,2,3-triazole conjugates with varied functional groups including benzyl, phenyl and heterocycle moieties was thus synthesized. Many of the designed derivatives showed potent antiplatelet aggregation activities with IC_50_ values of 5~21 nM.

## 1. Introduction

*Ginkgo biloba*, also named maidenhair tree, the only surviving species from the family Ginkgoaceae has existed for more than 180 million years, and for this reason it was called a “living fossil” by Darwin. *G. biloba* has been used as a traditional Chinese medicine for a long time for the treatment of lung weakness, asthma, coughing, cancer, etc [1,2]. Ginkgo has also been popular in the Western world since 1965, when a German company developed from ginkgo extracts a botanical medicine named EGB761, with various effects on central nervous system (CNS) diseases, including Alzheimer’s disease, dementia, hypomnesia, etc [3,4]. The main components of ginkgo extracts are terpene trilactones (including ginkgolides and bilobalide) and flavonoids [5,6]. Since flavonoids are deemed to hardly penetrate the blood-brain barrier, it is assumed the terpene trilactones from ginkgo extracts should be the major active components for the CNS effects and cardiovascular activity [7,8]. As a natural phospholipid agonist of the platelet activating factor of platelet activating factor receptor (PAFR), platelet activating factor (PAF) regulates various physiological activities of the CNS and peripheral nervous system, including platelet aggregation, blood pressure regulation, inflammation, long-term enhancement of CNS, etc [9]. It’s reported that ginkgolides could competitively inhibit the platelet-activating factor receptor (PAFR), resulting in the observed CNS protection and antithrombotic effects [10,11].

To date, 10 ginkgolides (ginkgolide A~Q) [5,6,12] and two bilobalides (bilobalide and bilobanol) [13,14] have been isolated and from *G. biloba* and their structures elucidated. Most natural ginkgolides displayed significant activity against PAFR, while the bilobalides didn’t. In particular, ginkgolide B is the most potent PAFR antagonist discovered in nature [1]. Since the 1970s, many investigations on the structural modifications and structure-activity relationship of ginkgolides have been conducted. It is revealed that both ring C and ring D are essential for anti-PAFR activity [15]. Substituents at the C-7 position decrease the activity [16]. It’s noteworthy that the introduction of bulky or aromatic substituents at 10-*O*H could help to increase activity against PAFR [17,18]. The 1,2,3-triazole moiety, also known simply as triazole, acts as an important structural fragment widely used to construct new drug molecules [19]. The triazole is an electron isostere of the amide group, which easily forms hydrogen bonds, coordination bonds, etc., helping to form a variety of non-covalent bond interactions with target proteins [19,20].

In this paper, a series of ginkgolide derivatives with 1,2,3-triazole moieties connected with various benzyl, phenyl and heterocycle moieties at the C-10 position were designed and synthetized. Their antiplatelet aggregation activities were also evaluated and several derivatives displayed more potent inhibitory effects against PAFR than the natural ginkgolide B, with IC_50_ values of 5~21 nM, or about 10 to 20 times higher than the natural compound.

## 2. Results and Discussion

### 2.1. Chemistry

Drugability is improved when the triazole moiety is introduced into some leads [20]. The azide-alkyne Huisgen cycloaddition reaction, also known as the Huisgen 1,3-dipolar cycloaddition, has been proved to be a powerful tool in construction of triazoles [21]. In this reaction, the azide moiety reacts to a terminal alkyne group to form the triazole ring. The copper(I) catalyst improves both the reaction rate and selectivity. A series of ginkgolide-1,2,3-triazole conjugates with varied functional groups including benzyl, phenyl and heterocycle moieties was synthesized via this method.

**Scheme 1:**
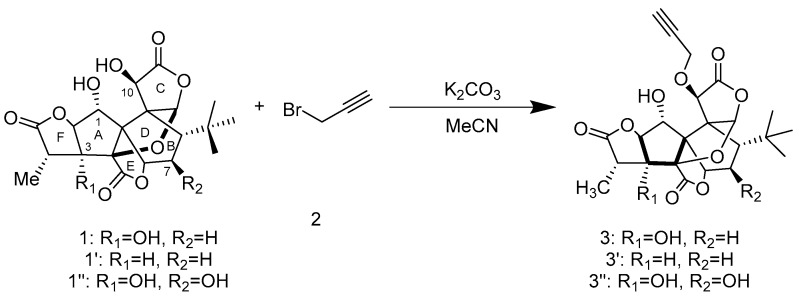
Formation of 10-*O*-propargylated ginkgolides **3**, **3′** and **3″**.

10-*O*-Propargylated ginkgolide B (**3**) was synthesized in 65% yield by mixing ginkgolide B (**1**) and 3-bromoprop-1-yne (**2**) with potassium carbonate in anhydrous acetonitrile for several hours (Scheme 1). 10-*O*-Propargylated ginkgolide A (**3′**) and 10-*O*-propargylated ginkgolide C (**3″**) were obtained in 32% and 69% yield, respectively from the corresponding ginkgolide A (**1′**) and ginkgolide C (**1″**) following the same scheme.

**Scheme 2:**
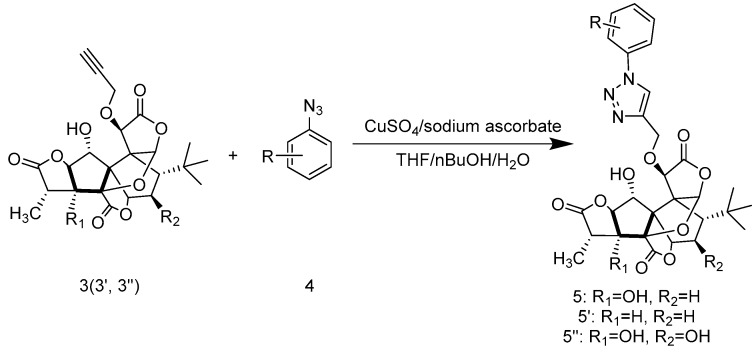
Formation of 10-substituted 1,2,3-triazole-ginkgolide derivatives **5**, **5′** and **5″**.

The target molecules, 10-substituted 1,2,3-triazole-ginkgolide B derivatives **5**, were synthesized using the copper(I)-catalyzed Huisgen 1,3-dipolar cycloaddition reaction [21] by mixing 10-*O*-propargylated ginkgolide B (**3**) with a series of azides **4** in a three-phase solvent, using copper(I) as catalyst (Scheme 2). The 1,2,3-triazole-ginkgolide A derivatives (**5′**) and 1,2,3-triazole-ginkgolide ginkgolide C derivatives (**5″**) were obtained by the same route.

**Scheme 3:**
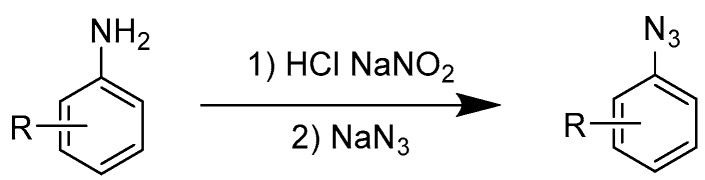
Preparation of azides **4a**–**4ee.**

Azides **4a**–**4ee** were synthesized by firstly mixing the corresponding aniline, sodium nitrite and concentrated hydrochloride in ethyl acetate at 0 °C for 30 min, then adding sodium azide to the system at room temperature and stirring for another two hours (Scheme 3) [22,23,24]. The resulting products are listed in Table 1.

**Scheme 4:**
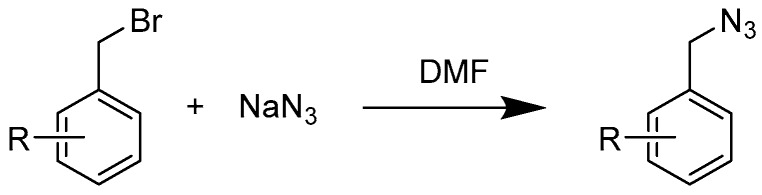
Preparation of azides **4ff**, **4gg**.

Azides **4ff**–**4gg** were synthesized by mixing the corresponding benzyl bromide and sodium azide in DMF for two hours (Scheme 4) [22,25]. The resulting products are listed in Table 2.

**Figure 1:**
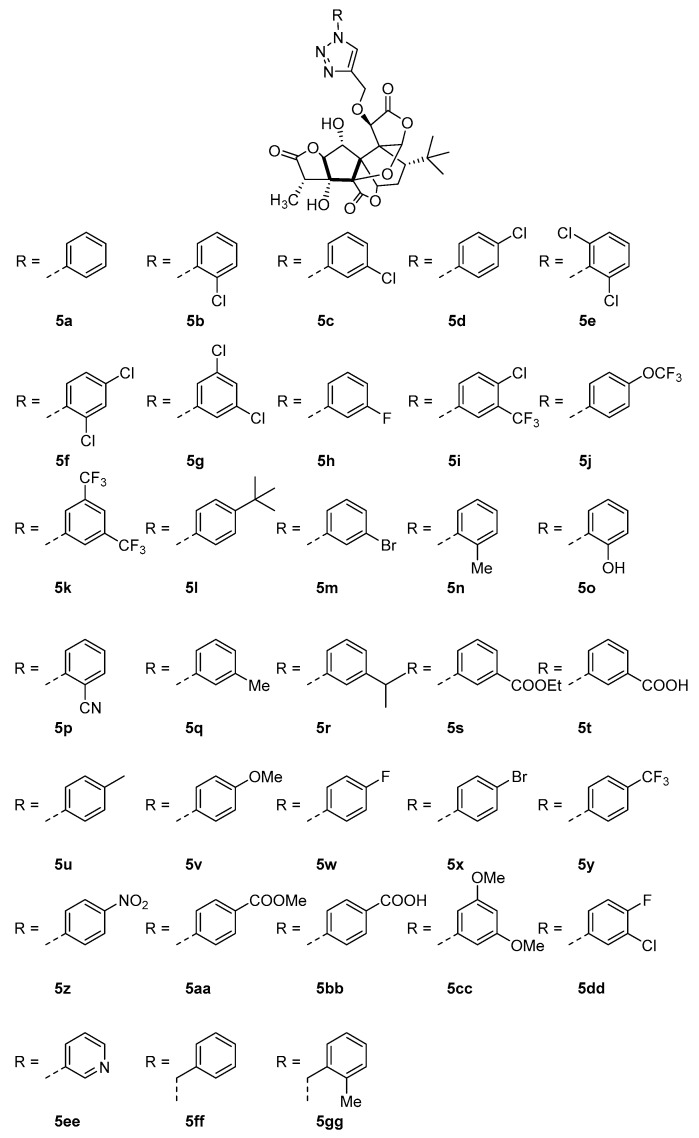
Structures of 10-substituted 1,2,3-triazole-ginkgolide B derivatives **5a**–**5gg**.

Following the reaction shown in Scheme 2, using 10-*O*-propargylated ginkgolide B **3** and azides **4a**-**4gg**, compounds **5a**-**5gg** were obtained (Figure 1).

**Figure 2:**
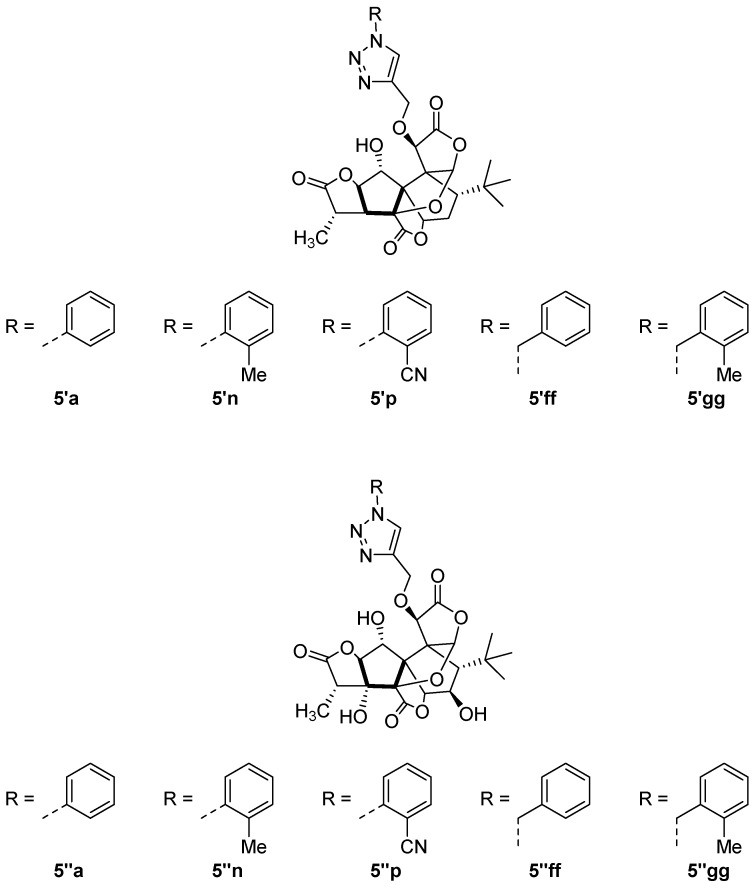
Structures of 10-substituted 1,2,3-triazole-ginkgolide A derivatives **5′a**-**5′gg** and 10-substituted 1,2,3-triazole-ginkgolide C derivatives **5″a**-**5″gg**.

Following the reaction shown in Scheme 2, using 10-*O*-propargylated ginkgolide A **3′**, 10-*O*-propargylated ginkgolide C **3″** and azides **4a**, **4n**, **4p**, **4ff**, **4gg**, compounds **5′a-5′gg**, **5″a-5″gg** were obtained as illustrated in Figure 2.

### 2.2. Biology

Since ginkgolide derivatives have shown antiplatelet aggregation activities as reported before [15,16,17,18], the as-synthesized ginkgolide-1,2,3-triazole derivatives were expected to show improved biological potency.

**Figure 3:**
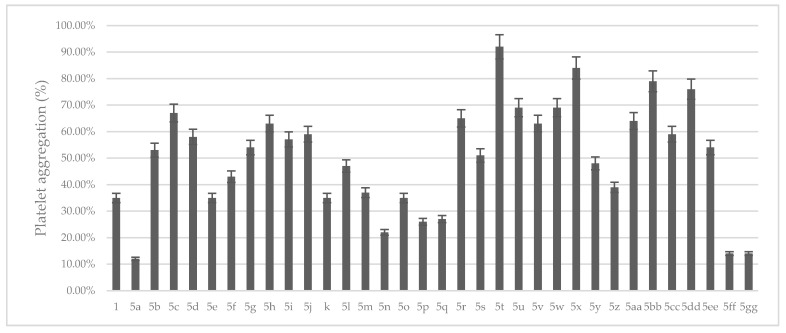
Antiplatelet aggregation activities of the 10-substituted 1,2,3-triazole-ginkgolide B derivatives. Ginkgolide B (**1**) was used as reference.

Firstly, the newly synthesized 10-substituted 1,2,3-triazole-ginkgolide B derivatives (**5**) were tested by the method of Born [26,27]. The result was given in Figure 3. Which showed some of them exhibited considerable activity better than ginkgolide B. The best results were obtained with compounds **5a**, **5n**, **5p**, **5ff** and **5gg**.

From the results shown above, we can preliminarily conclude that non-substituted benzyl (compound **5a**) and phenyl (compound **5ff**) 1,2,3-triazole conjugates have significantly enhanced antiplatelet aggregation activities compared to ginkgolide B (**1**). We can also see that the substitution at the *meta*- or/and *para*- positions of the benzyl group reduce the activity. Some small steric hindrance groups (such as methyl and cyano groups) substituted at the *ortho*-position of the phenyl (compounds **5n** and **5p**) and benzyl (compound **5gg**) groups maintain or slightly reduce the activity.

**Figure 4:**
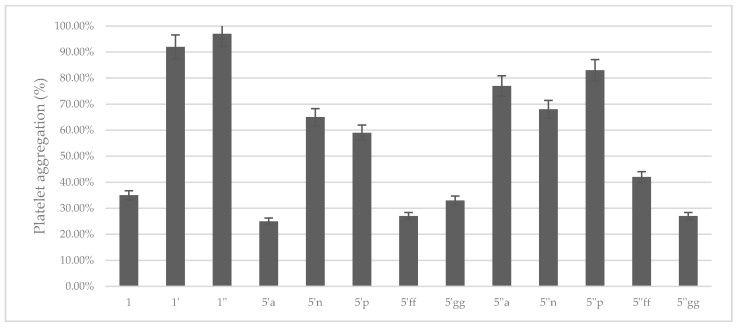
Antiplatelet aggregation activity activities of the 10-substituted 1,2,3-triazole-ginkgolide A and ginkgolide C derivatives. Ginkgolide B (**1**), ginkgolide A (**1′**) and ginkgolide C (**1″**) were used as reference.

In further studies, 10-substituted 1,2,3-triazole-ginkgolide A **5′** and ginkgolide C **5″** derivatives, having the same moieties as the most active ginkgolide B derivatives **5a**, **5n**, **5p**, **5ff** and **5gg**, were synthesized and their antiplatelet aggregation activities tested and reported as inhibition ratios at 50 nM). The results are shown in Figure 4. Some of them exhibit better activity than not only their precursors **1′** or **1″**, but also ginkgolide B (**1**). The best results were obtained with compounds **5′a**, **5′ff**, **5′gg** and **5″gg**.

The most active compounds obtained by method above, **5a**, **5n**, **5p**, **5q**, **5ff**, **5gg**, **5′a**, **5′ff**, **5′gg** and **5″gg**, were further examined in order to get their activity expressed as an IC_50_ value. The results are listed in Table 3.

As we can see, compounds **5a**, **5p**, **5ff**, **5gg** and **5′a** display promising antiplatelet aggregation activity with IC_50_ values ranging from 5–21 nM. Among them compounds **5ff** and **5gg** were the best among the series of compounds, showing about a 20-fold increase in comparison with the natural ginkgolide B (**1**).

**Figure 5:**
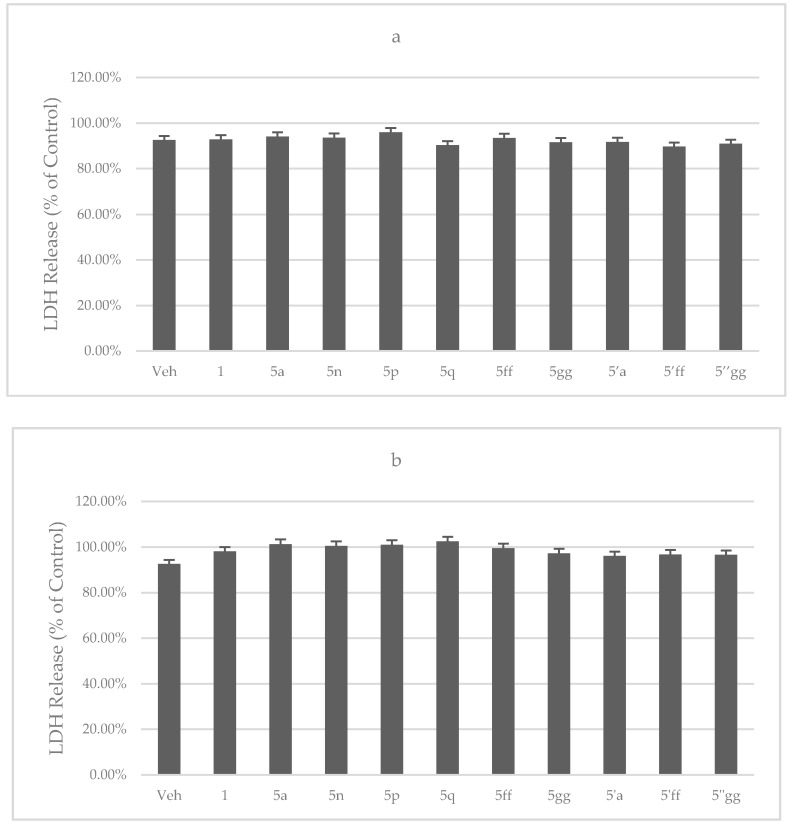
Cytotoxicity of some of ginkgolide-1,2,3-triazole derivatives: (**a**) Tested at 1 μM concentration. (**b**) Tested at 10 μM concentration. Ginkgolide B (**1**) was used as reference.

In order to verify if the most active ginkgolide-1,2,3-triazole derivatives could also be considered as potential antiplatelet aggregation therapeutics, compounds **5a**, **5n**, **5p**, **5q**, **5ff**, **5gg**, **5′a**, **5′ff** and **5″gg** were examined to confirm their cytotoxicity using an LDH assay [28]. The results are shown in Figure 5.

**Figure 6:**
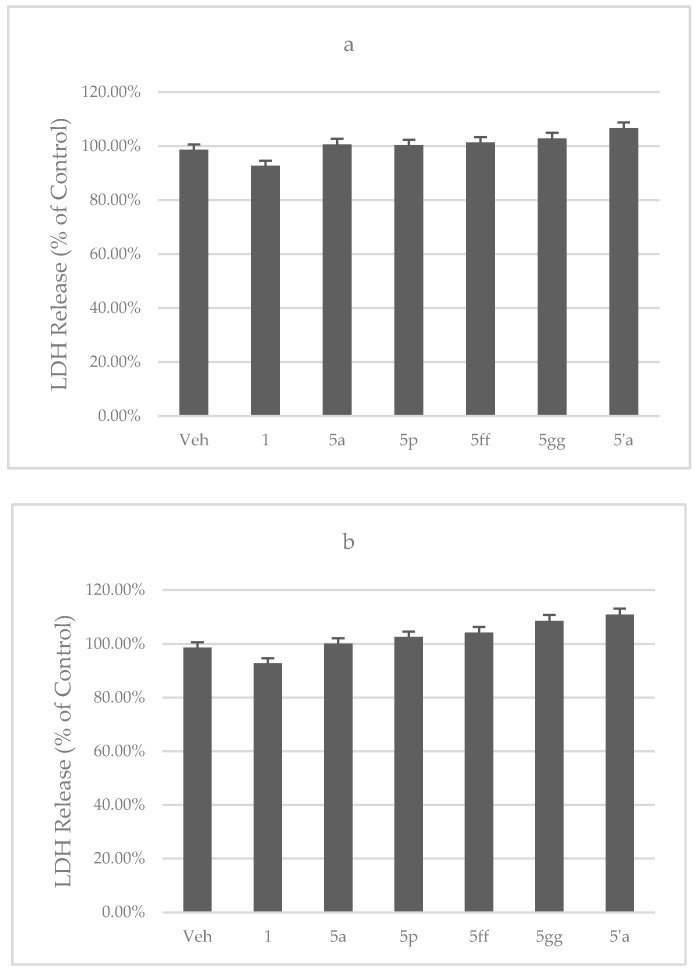
Toxicity on platelets of some of ginkgolide-1,2,3-triazole derivatives: (**a**) Tested at 1 μM concentration. (**b**) Tested at 10 μM concentration. Ginkgolide B (**1**) was used as reference.

In addition, compounds **5a**, **5p**, **5ff**, **5gg** and **5′a** were examined to confirm their toxicity on platelets using the LDH assay [29]. The results are shown in Figure 6.

As shown in results above, these most active compounds did not demonstrate toxicity towards cardiomyocytes and platelets (*p* > 0.05) up to 10 μM (almost two order of magnitude higher than IC_50_ of ginkgolide B), which suggest that they have a broad therapeutic window/safety window.

## 3. Materials and Methods

### 3.1. Compound Synthesis

#### 3.1.1. General Experimental Procedures

All solvents and reagents of analytical grade were obtained from commercial sources. Flash chromatography was performed using silica gel (200–300 mesh, Qingdao Marine Chemical Group Co., Qingdao, China). All reactions were monitored by TLC on silica gel plates (Merck, Darmstadt, Germany). NMR spectra were recorded in CDCl_3_ or DMSO at 400 or 600 MHz for ^1^H-NMR and 125 or 150 MHz for ^13^C-NMR on an Ascent 400 or 600 spectrometer (Bruker, Fallanden, Switzerland). The solvent signal was used as an internal standard. ESI-MS were recorded on an 1200/MSD mass spectrometer (Agilent, Santa Clara, CA, USA). HREIMS were recorded on a LTQ Orbitrap XL mass spectrometer (Thermo, Bremen, Germany).

#### 3.1.2. General Procedures for the Preparation 10-*O*-propargylated Ginkgolides

Propargyl bromide (2.4 mmol) were slowly added to a mixture of ginkgolide (**1**, **1′** or **1″**, 2.0 mmol) and K_2_CO_3_ (4.0 mmol) in acetonitrile (15 mL). The reaction mixture was refluxed for 24 h under an argon atmosphere and then was extracted with EtOAc three times. The combined organic phases were dried over anhydrous MgSO_4_, filtered, and concentrated in vacuo. The residue was purified by chromatography (SiO_2_, petroleum ether (PE)/EtOAc stepwise elution, 1:1 to EtOAc).

*10*-*O-Propargylated ginkgolide B* (**3**). Following the described procedure, 602 mg (65%) of compound **3** were obtained from 829 mg (2.0 mmol) of ginkgolide B (**1**). ^1^H-NMR (600 MHz, DMSO-*d*_6_) δ 6.46 (s, 1H), 6.14 (s, 1H), 5.32 (d, *J* = 4.1 Hz, 1H), 5.19 (s, 1H), 5.15 (d, *J* = 4.9 Hz, 1H), 4.69 (ddd, *J* = 70.7, 15.8, 2.5 Hz, 2H), 4.61 (d, *J* = 6.8 Hz, 1H), 4.09 (dd, *J* = 6.9, 4.9 Hz, 1H), 3.62 (t, *J* = 2.4 Hz, 1H), 2.84 (q, *J* = 7.1 Hz, 1H), 2.13 (dd, *J* = 13.6, 4.6 Hz, 1H), 1.99 (s, 1H), 1.87 (td, *J* = 13.9, 4.2 Hz, 1H), 1.71 (dd, *J* = 14.4, 4.5 Hz, 1H), 1.11 (d, *J* = 7.2 Hz, 3H), 1.05 (s, 9H). ^13^C-NMR (150 MHz, DMSO-*d*_6_) δ 176.77, 172.56, 170.63, 110.02, 99.51, 93.37, 83.06, 79.71, 79.29, 74.96, 74.12, 72.41, 67.84, 60.23, 57.91, 49.08, 41.96, 36.96, 32.29, 29.38 (3C), 8.44. HRMS (ESI): *m*/*z* calcd for C_23_H_26_O_10_ [M + H]^+^: 463.1599, found 463.1531.

*10*-*O-Propargylated ginkgolide A* (**3′**), Following the described procedure, 286 mg (32%) of compound **3′** were obtained from 797 mg (2.0 mmol) of ginkgolide A (**1′**). ^1^H-NMR (600 MHz, DMSO-*d*_6_) δ 6.43 (s, 1H), 6.12 (s, 1H), 5.10 (s, 1H), 5.00 (d, *J* = 4.1 Hz, 1H), 4.86 (t, *J* = 7.7 Hz, 1H), 4.72 (dd, *J* = 15.6, 2.5 Hz, 1H), 4.50 (dd, *J* = 15.5, 2.5 Hz, 1H), 3.59 (s, 1H), 3.17 (d, *J* = 5.2 Hz, 1H), 2.91 (q, *J* = 7.2 Hz, 1H), 2.75 (dd, *J* = 15.1, 7.2 Hz, 1H), 2.08 (dd, *J* = 13.7, 4.8 Hz, 1H), 1.99 (s, 1H), 1.95 (dd, *J* = 13.8, 4.3 Hz, 1H), 1.82 (dd, *J* = 15.1, 8.2 Hz, 1H), 1.72 (dd, *J* = 14.1, 4.8 Hz, 1H), 1.23 (s, 2H), 1.12 (d, *J* = 7.2 Hz, 3H), 1.03 (s, 9H). ^13^C-NMR (150 MHz, DMSO-*d*_6_) δ 176.91,172.75, 170.96, 110.08, 100.94, 87.95, 86.48, 85.31, 79.49, 78.95, 75.32, 68.67, 66.83, 57.81, 49.07, 49.03, 36.78, 36.45, 32.25, 29.24 (3C), 8.64. HRMS (ESI): *m*/*z* calcd for C_23_H_26_O_9_ [M + H]+: 447.1650, found 447.1582.

*10*-*O-Propargylated ginkgolide C* (**3″**). Following the described procedure, 669 mg (69%) of compound **3″** were obtained from 861 mg (2.0 mmol) of ginkgolide A (**1″**). ^1^H-NMR (600 MHz, DMSO-*d*_6_) δ 6.47 (s, 1H), 6.17 (s, 1H), 5.68 (d, *J* = 6.1 Hz, 1H), 5.26 (d, *J* = 5.0 Hz, 1H), 5.18 (s, 1H), 4.71 (ddd, *J* = 67.5, 15.8, 2.5 Hz, 2H), 4.59 (d, *J* = 6.6 Hz, 1H), 4.06–4.01 (m, 2H), 3.99 (ddd, *J* = 12.6 Hz, 6.1 Hz, 4.3 Hz, 1H), 3.63 (s, 1H), 2.82 (q, *J* = 7.1 Hz, 1H), 2.50 (s, 2H), 1.99 (s, 2H), 1.54 (d, *J* = 12.5 Hz, 1H), 1.18 (t, *J* = 7.1 Hz, 2H), 1.12 (s, 9H). ^13^C-NMR (150 MHz, DMSO-*d*_6_) δ 176.73, 172.50, 170.90, 109.86, 99.26, 93.45, 83.06, 79.82, 79.29, 74.79, 74.17, 73.95, 67.05, 64.01, 60.23, 58.01, 49.26, 41.94, 32.20, 21.23 (3C), 8.48. HRMS (ESI): *m*/*z* calcd for C_23_H_26_O_11_ [M + H]^+^: 479.1548, found 479.1472.

#### 3.1.3. General Procedures for the Preparation of 10-Substituted 1,2,3-triazole-Ginkgolide Derivatives

Sodium ascorbate (0.03 mmol) and CuSO_4_ (0.01 mmol) were added in single portions to a solution of 10-*O*-propargylated ginkgolide **3**, **3′** or **3″** (0.1 mmol) and corresponding azide **4** (0.11 mmol) in 1:1:1 t-BuOH/ H_2_O/THF (3 mL). The reaction mixture was stirred at room temperature for 48 h under an argon atmosphere and then was extracted with EtOAc three times. The combined organic phases were dried over anhydrous MgSO_4_, filtered, and concentrated in vacuo. The residue was purified by chromatography (SiO_2_, PE/EtOAc 1:3 stepwise elution to EtOAc) to afford the appropriate compound **5**.

*10*-*O-(1-Phenyl-1H-1,2,3-triazole) ginkgolide B* (**5a**). Following the described procedure, 42.3 mg (73%) of compound **5a** were obtained from 46.2 mg (0.1 mmol) of 10-*O*-propargylated ginkgolide B (**3**). ^1^H-NMR (400 MHz, DMSO-*d*_6_) δ 8.798 (1H, s), 7.88 (d, *J* = 7.6 Hz, 2H), 7.50–7.64 (m, 3H), 6.47 (s, 1H), 6.20 (s, 1H), 5.52 (d, *J* = 4.4 Hz, 1H), 5.45 (d, *J* = 12.0 Hz, 1H), 4.93 (d, *J* = 12.0 Hz, 1H), 4.63 (d, *J* = 6.8 Hz, 1H), 4.21 (dd, *J* = 6.8 Hz, 4.8 Hz, 1H), 2.88 (q, *J* = 6.8 Hz, 1H), 2.11 (dd, *J* = 13.6 Hz, 4.0 Hz, 1H), 1.84 (ddd, *J* = 14.0 Hz, 13.6 Hz, 4.0 Hz, 1H), 1.73 (dd, *J* =14.4 Hz, 4.0 Hz, 1H), 1.13 (d, *J* = 7.2 Hz, 3H), 1.02 (s, 9H). ^13^C-NMR (125 MHz, DMSO-*d*_6_) δ 176.32, 172.51, 170.12, 143.76, 136.46, 129.96 (2C), 128.94, 121.59, 120.23 (2C), 109.68, 98.88, 92.62, 82.47, 78.49, 75.16, 73.77, 71.96, 67.34, 63.00, 48.73, 41.56, 36.54, 31.73, 28.64 (3C), 7.84. HRMS (ESI): *m*/*z* calcd for C_29_H_31_N_3_O_10_ [M + H]^+^: 582.2082, found 582.2062.

*10*-*O-(1-(2-Chlorophenyl)-1H-1,2,3-triazole) ginkgolide B* (**5b**). Following the described procedure, 21.0 mg (34%) of compound **5b** were obtained from 46.2 mg (0.1 mmol) of 10-*O*-propargylated ginkgolide B (**3**). ^1^H-NMR (400 MHz, DMSO-*d*_6_) δ 7.78 (d, *J* = 1.2 Hz, 1H), 7.58–7.71 (m,2H), 6.47 (s, 1H), 6.20 (s, 1H), 5.50 (d, *J* = 4.8 Hz, 1H), 5.46 (d, *J* = 12.0 Hz, 1H), 5.33–5.35 (m, 2H), 4.97 (d, *J* = 12.0 Hz, 1H), 4.64 (d, *J* = 7.2 Hz, 1H), 4.21 (dd, *J* = 7.2 Hz, 4.8 Hz, 1H), 2.88 (q, *J* = 7.0 Hz, 1H), 2.12 (dd, *J* = 13.6 Hz, 4.0 Hz, 1H), 1.85 (ddd, *J* = 14.4 Hz, 13.4 Hz, 4.0 Hz, 1H), 1.74 (dd, *J* = 14.4 Hz, 4.0 Hz,1H), 1.12 (d, *J* = 6.8 Hz, 3H), 1.02 (s, 9H), 176.30, 172.47, 170.10, 142.91, 134.30, 131.77, 130.60, 128.52, 128.39, 128.31, 125.43, 109.67, 98.86, 92.58, 82.46, 78.47, 75.25, 73.74, 71.93, 67.32, 63.07, 48.72, 41.55, 36.51, 31.72, 28.65 (3C), 8.57 (s,1H). ^13^C-NMR (125 MHz, DMSO-d6) δ7.82, 7.80 (d, *J* = 1.2 Hz, 1H). HRMS (ESI): *m*/*z* calcd for C_29_H_30_ClN_3_O_10_ [M + H]^+^: 616.1692, found 616.1670.

*10*-*O-(1-(3-Chlorophenyl)-1H-1,2,3-triazole) ginkgolide B* (**5c**). Following the described procedure, 40.1 mg (65%) of compound **5c** were obtained as method 1, from 46.2 mg (0.1 mmol) of 10-*O*-propargylated ginkgolide B (**3**).^1^H-NMR (400 MHz, DMSO-*d*_6_) δ 8.87 (s, 1H), 8.02 (s, 1H), 7.59–7.91 (m, 3H), 6.47 (s, 1H), 6.20 (s, 1H), 5.43–5.46 (m, 2H), 5.33 (s, 2H), 4.93 (d, *J* = 12.0 Hz, 1H), 4.64 (d, *J* = 6.8 Hz, 1H), 4.20 (dd, *J* = 7.0 Hz, 4.6 Hz, 1H), 2.88 (q, *J* = 6.8 Hz, 1H), 2.11 (dd, *J* = 13.0 Hz, 3.8 Hz, 1H), 1.86 (ddd, *J* = 14.2 Hz, 13.4 Hz, 3.8 Hz, 1H), 1.73 (dd, *J* = 14.4 Hz, 4.0 Hz, 1H), 1.12(d, *J* = 6.8 Hz, 3H), 1.02 (s, 9H). ^13^C-NMR (125 MHz, DMSO-*d*_6_) δ 176.28, 172.46, 170.08, 143.82, 137.48, 134.20, 131.69, 128.74, 121.91, 120.02, 118.84, 109.66, 98.88, 92.60, 82.47, 78.45, 75.14, 73.75, 71.93, 67.29, 62.90, 48.71, 41.53, 36.51, 31.70, 28.62 (3C), 7.82. HRMS (ESI): *m*/*z* calcd for C_29_H_30_ClN_3_O_10_ [M + H]+: 616.1692, found 616.1674.

*10*-*O-(1-(4-Chlorophenyl)-1H-1,2,3-triazole) ginkgolide B* (**5d**). Following the described procedure, 20.3 mg (33%) of compound **5d** were obtained from 46.2 mg (0.1 mmol) of 10-*O*-propargylated ginkgolide B (**3**). ^1^H-NMR (400 MHz, DMSO-*d*_6_) δ8.82 (s,1H), 7.68–7.94 (dd, 4H), 6.47 (s, 1H), 6.20 (s, 1H), 5.43–5.48 (m, 2H), 5.32–5.33 (m, 2H), 4.92 (d, *J* = 8.0 Hz, 1H), 4.63 (d, *J* = 7.2 Hz, 1H), 4.20 (dd, *J* = 7.0 Hz, 4.6 Hz, 1H), 2.88 (q, *J* = 6.8 Hz, 1H), 2.11 (dd, *J* = 13.0 Hz, 3.8 Hz, 1H), 1.83 (ddd, *J* = 14.4 Hz, 13.2Hz, 4.0 Hz, 1H), 1.73 (dd, *J* = 14.4 Hz, 4.0 Hz, 1H), 1.13 (d, *J* = 7.2 Hz, 3H), 1.02 (s, 9H). ^13^C-NMR (125 MHz, DMSO-*d*_6_) δ 176.28, 172.46, 170.08, 143.87, 135.24, 133.19, 129.91 (2C), 121.90 (2C), 121.73, 109.66, 98.87, 92.60, 82.46, 78.45, 75.16, 73.74, 71.92, 67.30, 62.93, 48.70, 41.53, 36.51, 31.70, 28.62 (3C), 7.81. HRMS (ESI): *m*/*z* calcd for C_29_H_30_ClN_3_O_10_ [M + H]+: 616.1692, found 616.1671.

*10*-*O-(1-(2,6-Dichlorophenyl)-1H-1,2,3-triazole) ginkgolide B* (**5e**). Following the described procedure, 34.5 mg (53%) of compound **5e** were obtained from 46.2 mg (0.1 mmol) of 10-*O*-propargylated ginkgolide B (**3**). ^1^H-NMR (400 MHz, DMSO-*d*_6_) δ8.54 (s,1H), 7.68–7.81(m, 3H), 6.48 (s, 1H), 6.20 (s, 1H), 5.47 (d, *J* = 12.0 Hz, 1H), 5.32–5.54 (m, 2H), 5.24 (d, *J* = 4.4 Hz, 1H), 4.99 (d, *J* = 12.4 Hz, 1H), 4.64 (d, *J* = 7.2 Hz, 1H), 4.19 (dd, *J* = 7.2 Hz, 4.6 Hz, 1H), 2.87 (q, *J* = 7.2 Hz, 1H), 2.13 (dd, *J* = 13.2 Hz, 4.0 Hz, 1H), 1.84 (ddd, *J* = 14.2 Hz, 13.4 Hz, 4.0 Hz, 1H), 1.74 (dd, *J* = 14.4 Hz, 4.0 Hz, 1H), 1.12 (d, *J* = 7.2 Hz, 3H), 1.03 (s, 9H). ^13^C-NMR (125 MHz, DMSO-*d*_6_) δ 176.27, 172.38, 170.07, 143.11, 133.04, 132.58 (2C), 132.42, 129.26 (2C), 125.88, 109.68, 98.82, 92.40, 82.48, 78.45, 75.51, 73.74, 71.91, 67.28, 63.20, 48.68, 41.54, 36.50, 31.74, 28.69 (3C), 7.80. HRMS (ESI): *m*/*z* calcd for C_29_H_29_Cl_2_N_3_O_10_ [M + H]^+^: 650.1303, found 650.1287.

*10*-*O-(1-(2,4-Dichlorophenyl)-1H-1,2,3-triazole) ginkgolide B* (**5f**). Following the described procedure, 29.3 mg (45%) of compound **5f** were obtained from 46.2 mg (0.1 mmol) of 10-*O*-propargylated ginkgolide B (**3**). ^1^H-NMR (400 MHz, DMSO-*d*_6_) δ 8.57 (s,1H), 8.02(d, *J* = 2.4 Hz, 1H), 7.68–7.76(m, 2H), 6.47 (s, 1H), 6.20 (s, 1H), 5.43–5.48 (m, 2H), 5.33–5.40 (m, 2H), 4.96 (d, *J* = 12.4 Hz, 1H), 4.63 (d, *J* = 7.2 Hz, 1H), 4.20 (dd, *J* = 7.2 Hz, 4.8Hz, 1H), 2.88 (q, *J* = 6.8 Hz, 1H), 2.12 (dd, *J* = 12.8 Hz, 4.0 Hz, 1H), 1.84 (ddd, *J* = 14.2 Hz, 13.2 Hz, 4.0 Hz, 1H), 1.74 (dd, *J* = 14.4 Hz, 4.0 Hz, 1H), 1.12(d, *J* = 6.8 Hz, 3H), 1.02 (s, 9H).^13^C-NMR (125 MHz, DMSO-*d*_6_) δ 176.27, 172.44, 170.07, 143.02, 135.51, 133.34, 130.16, 129.64, 129.49, 128.65, 125.51, 109.65, 98.85, 92.58, 82.45, 78.44, 75.27, 73.71, 71.91, 67.30, 63.03, 59.71, 48.69, 41.53, 36.49, 31.71, 28.64 (3C), 7.81. HRMS (ESI): *m*/*z* calcd for C_29_H_29_Cl_2_N_3_O_10_ [M + H]^+^: 650.1303, found 650.1289.

*10*-*O-(1-(3,5-Dichlorophenyl)-1H-1,2,3-triazole) ginkgolide B* (**5g**). Following the described procedure, 38.3 mg (34%) of compound **5g** were obtained from 46.2 mg (0.1 mmol) of 10-*O*-propargylated ginkgolide B (**3**). ^1^H-NMR (400 MHz, DMSO-*d*_6_) δ 8.92 (s,1H), 8.05 (d, *J* = 1.6 Hz, 2H), 7.80 (t, *J* = 1.6 Hz, 1H), 6.48 (s, 1H), 6.20 (s, 1H), 5.44 (d, *J* = 12.0 Hz,1H), 5.36 (d, *J* = 4.8 Hz, 1H), 5.32–5.35 (m, 2H), 4.93 (d, *J* = 12.0 Hz, 1H), 4.63 (d, *J* = 7.2 Hz, 1H), 4.20 (dd, *J* = 7.2 Hz, 4.6Hz, 1H), 2.87 (q, *J* = 7.2 Hz, 1H), 2.11 (dd, *J* = 12.8 Hz, 4.0 Hz, 1H), 1.83 (ddd, *J* = 14.4 Hz, 13.2 Hz, 4.0 Hz, 1H), 1.73 (dd, *J* = 14.4 Hz, 4.0 Hz, 1H), 1.12 (d, *J* = 7.2 Hz, 3H), 1.02 (s, 9H). ^13^C-NMR (125 MHz, DMSO-*d*_6_) δ 176.28, 172.43, 170.08, 143.90, 138.05, 135.26 (2C), 128.27, 122.22, 118.98, 118.88, 109.66, 98.91, 92.60, 82.49, 78.44, 75.17, 73.75, 71.93, 67.28, 62.84, 48.71, 41.52, 36.51, 31.71, 28.62 (3C), 7.84. HRMS (ESI): *m*/*z* calcd for C_29_H_29_Cl_2_N_3_O_10_ [M + H]^+^: 650.1303, found 650.1287.

*10*-*O-(1-(3-Fluorophenyl)-1H-1,2,3-triazole) ginkgolide B* (**5h**). Following the described procedure, 32.4 mg (54%) of compound **5h** were obtained from 46.2 mg (0.1 mmol) of 10-*O*-propargylated ginkgolide B (**3**).^1^H-NMR (400 MHz, DMSO-*d*_6_) δ 7.99 (s, 1H), 7.16–7.54 (m, 4H), 6.01 (s, 1H), 5.67 (d, *J* = 11.6 Hz, 1H), 5.53 (d, *J* = 3.6 Hz, 1H), 5.05 (d, *J* = 4.4 Hz, 1H), 4.96 (s, 1H), 4.91 (d, *J* = 11.2 Hz, 1H), 4.64 (d, *J* = 7.6 Hz, 1H), 4.40 (dd, *J* = 7.6 Hz, 4.4 Hz, 1H), 3.07 (q, *J* = 6.8 Hz, 1H), 2.84 (s, 1H), 2.28 (dd, *J* = 12.8 Hz, 4.2 Hz, 1H), 2.05 (ddd, *J* = 16.8 Hz, 10.8 Hz, 4.0 Hz, 1H), 1.98 (dd, *J* = 14.2 Hz, 4.2 Hz, 1H), 1.30 (d, *J* = 6.8 Hz, 3H), 1.13 (s, 9H). ^13^C-NMR (125 MHz, DMSO-*d*_6_) δ 176.28, 172.46, 170.08, 143.86, 137.64, 131.93, 121.85, 116.14, 115.66, 109.66, 107.83, 107.62, 92.60, 82.47, 78.45, 75.17, 73.75, 71.93, 67.30, 62.92, 48.71, 41.53, 36.51, 31.71, 28.62 (3C), 7.82. HRMS (ESI): *m*/*z* calcd for C_29_H_31_FN_3_O_10_ [M + H]^+^: 600.1993, found 600.2014.

*10*-*O-(1-(4-Chloro-3-trifluoromethylphenyl)-1H-1,2,3-triazole) ginkgolide B* (**5i**). Following the described procedure, 27.9 mg (41%) of compound **5i** were obtained from 46.2 mg (0.1 mmol) of 10-*O*-propargylated ginkgolide B (**3**). ^1^H-NMR (400 MHz, DMSO-*d*_6_) δ 8.07 (s,1H), 7.70–8.09 (m, 3H), 6.01 (s, 1H,), 5.65 (d, *J* = 11.6 Hz, 1H), 5.52 (d, *J* = 4.0 Hz, 1H), 5.06 (d, *J* = 4.0 Hz, 1H), 4.97 (s, 1H), 4.91 (d, *J* = 11.6 Hz, 1H), 4.62 (d, *J* = 8.0 Hz, 1H), 4.38 (dd, *J* = 7.6 Hz, 4.4 Hz, 1H), 3.07 (q, *J* = 7.0 Hz, 1H), 2.98 (s, 1H), 2.28 (dd, *J* = 12.8 Hz, 4.0 Hz, 1H), 2.04 (ddd, *J* = 14.0 Hz, 13.2 Hz, 4.0 Hz, 1H), 1.95 (dd, *J* = 14.4 Hz, 4.0 Hz, 1H), 1.30 (d, *J* = 6.8 Hz, 3H), 1.12 (s, 9H). ^13^C-NMR (125 MHz, DMSO-*d*_6_) δ 176.27, 172.44, 170.07, 143.99, 135.40, 133.39, 130.69, 128.15, 127.83, 125.51, 122.25, 119.60, 109.66, 98.88, 92.56, 82.47, 78.43, 75.19, 73.74, 71.93, 67.28, 62.84, 48.70, 41.53, 36.50, 31.70, 28.62 (3C), 7.82. HRMS (ESI): *m*/*z* calcd for C_30_H_29_ClF_3_N_3_O_10_ [M + H]^+^: 684.1566, found 684.1544.

*10*-*O-(1-(4-Trifluoromethoxyphenyl)-1H-1,2,3-triazole) ginkgolide B* (**5j**). Following the described procedure, 20.6 mg (31%) of compound **5j** were obtained from 46.2 mg (0.1 mmol) of 10-*O*-propargylated ginkgolide B (**3**). ^1^H-NMR (400 MHz, DMSO-*d*_6_) δ 8.83 (s,1H), 8.03 (d, *J* = 9.0 Hz, 2H), 7.65 (d, *J* = 8.4 Hz, 2H), 6.47 (s, 1H), 6.20 (s, 1H), 5.43–5.49 (m, 2H), 5.32–5.34 (m, 2H), 4.93 (d, *J* = 12.0 Hz, 1H), 4.62 (d, *J* = 7.2 Hz, 1H), 4.21 (dd, *J* = 7.2 Hz, 4.6 Hz, 1H), 2.88 (q, *J* = 7.0 Hz, 1H), 2.11 (dd, *J* = 13.2 Hz, 4.0 Hz, 1H), 1.84 (ddd, *J* = 14.2 Hz, 13.4 Hz, 4.0 Hz, 1H), 1.73 (dd, *J* = 14.4 Hz, 4.0 Hz, 1H), 1.13(d, *J* = 7.2 Hz, 3H), 1.02 (s, 9H). ^13^C-NMR (125 MHz, DMSO-*d*_6_) δ 176.28, 172.46, 170.08, 148.02, 143.91, 135.30, 122.63 (2C), 122.30 (2C), 121.97, 121.00, 109.66, 98.87, 92.61, 82.46, 78.45, 75.17, 73.74, 71.93, 67.30, 62.94, 48.71, 41.53, 36.51, 31.70, 28.62 (3C), 7.81. HRMS (ESI): *m*/*z* calcd for C_30_H_30_F_3_N_3_O_11_ [M + H]^+^: 666.1905, found 666.1887.

*10*-*O-(1-(3,5-Ditrifluoromethylphenyl)-1H-1,2,3-triazole) ginkgolide B* (**5k**). Following the described procedure, 32.3 mg (45%) of compound **5k** were obtained from 46.2 mg (0.1 mmol) of 10-*O*-propargylated ginkgolide B (**3**). ^1^H-NMR (400 MHz, DMSO-*d*_6_) δ 9.14 (s, 1H), 8.62 (s, 2H), 8.31 (s, 1H), 6.48 (s, 1H), 6.21 (s, 1H), 5.47 (d, *J* = 12.0 Hz, 1H), 5.35 (s, 1H), 5.30–5.33 (m, 2H), 4.96 (d, *J* = 12.0 Hz, 1H), 4.64 (d, *J* = 7.2 Hz, 1H), 4.21 (dd, *J* = 7.2 Hz, 4.8 Hz, 1H), 2.88 (q, *J* = 6.8 Hz, 1H), 2.12 (dd, *J* = 12.8 Hz, 4.0 Hz,1H), 1.71–1.87 (m, 2H), 1.13 (d, *J* = 6.8 Hz, 3H), 1.03 (s, 9H). ^13^C-NMR (125 MHz, DMSO-*d*_6_) δ 176.28, 172.44, 170.07, 144.01, 137.73, 132.00 (2C), 131.73 (2C), 123.81, 122.64, 121.64, 121.07, 109.68, 98.90, 92.54, 82.49, 78.42, 75.22, 73.77, 71.94, 62.78, 48.71, 41.53, 36.50, 31.71, 28.63 (3C), 7.83. HRMS (ESI): *m*/*z* calcd for C_31_H_29_F_6_N_3_O_10_ [M + H]^+^: 718.1830, found 718.1808.

*10*-*O-(1-(4-tert-Butylphenyl)-1H-1,2,3-triazole) ginkgolide B* (**5l**). Following the described procedure, 35.0 mg (55%) of compound **5l** were obtained from 46.2 mg (0.1 mmol) of 10-*O*-propargylated ginkgolide B (**3**). ^1^H-NMR (400 MHz, DMSO-*d*_6_) δ 8.75 (s, 1H), 7.78 (d, *J* = 8.4 Hz, 2H), 7.62 (d, *J* = 8.8 Hz, 2H), 6.47 (s, 1H), 6.20 (s, 1H), 5.58 (d, *J* = 4.4 Hz, 1H), 5.44 (d, *J* = 12.0 Hz, 1H), 5.33 (s, 2H), 4.93 (d, *J* = 12.0 Hz, 1H), 4.63 (d, *J* = 7.2 Hz, 1H), 4.21 (dd, *J* = 7.2 Hz, 4.6 Hz, 1H), 2.88 (q, *J* = 7.2 Hz, 1H), 2.11 (dd, *J* = 13.2 Hz, 4.0 Hz, 1H), 1.83 (ddd, *J* = 14.4 Hz, 13.2 Hz, 4.0 Hz, 1H), 1.73 (dd, *J* = 14.4 Hz, 3.8 Hz, 1H), 1.33 (s, 9H), 1.13 (d, *J* = 7.2 Hz, 3H), 1.01 (s, 9H). ^13^C-NMR (125 MHz, DMSO-*d*_6_) δ 176.29, 172.50, 170.08, 150.60, 143.60, 134.13, 126.64 (2C), 121.49, 119.98 (2C), 109.65, 98.84, 92.60, 82.43, 78.46, 75.09, 73.73, 71.93, 67.32, 62.97, 48.71, 41.54, 36.51, 34.50, 31.70, 30.95 (3C), 28.62 (3C), 7.80. HRMS (ESI): *m*/*z* calcd for C_33_H_39_N_3_O_10_ [M + H]^+^: 638.2708, found 638.2689.

*10*-*O-(1-(3-Bromophenyl)-1H-1,2,3-triazole) ginkgolide B* (**5m**). Following the described procedure, 38.3 mg (58%) of compound **5m** were obtained from 46.2 mg (0.1 mmol) of 10-*O*-propargylated ginkgolide B (**3**). ^1^H-NMR (400 MHz, DMSO-*d*_6_) δ 8.87 (s, 1H), 8.14 (t, *J* = 2.0 Hz, 1H), 7.94 (dd, *J* = 8.2 Hz, 1.4 Hz, 1H), 7.73 (d, *J* = 8.4 Hz, 1H), 7.58 (t, *J* = 8.0 Hz, 1H), 6.47 (s, 1H), 6.20 (s, 1H), 5.43–5.46 (m, 2H), 5.33 (s, 2H), 4.93 (d, *J* = 12.0 Hz, 1H), 4.63 (d, *J* = 7.2 Hz, 1H), 4.20 (dd, *J* = 7.2 Hz, 4.6 Hz, 1H), 2.88 (q, *J* = 7.2 Hz, 1H), 2.11 (dd, *J* = 13.2 Hz, 4.0 Hz, 1H), 1.83 (ddd, *J* = 14.4 Hz, 13.2 Hz, 4.0 Hz, 1H), 1.73 (dd, *J* = 14.4 Hz, 4.2 Hz, 1H), 1.12 (d, *J* = 7.2 Hz, 3H), 1.01 (s, 9H). ^13^C-NMR (125 MHz, DMSO-*d*_6_) δ 176.28, 172.46, 170.08, 143.79, 137.55, 131.90, 131.66, 122.77, 122.43, 121.92, 119.23, 109.66, 98.88, 92.60, 82.47, 78.45, 75.13, 73.75, 71.93, 67.29, 62.89, 48.71, 41.53, 36.51, 31.70, 28.62 (3C), 7.83. HRMS (ESI): *m*/*z* calcd for C_29_H_31_BrN_3_O_10_ [M + H]^+^: 660.1193, found 660.1207.

*10*-*O-(1-(2-Methylphenyl)-1H-1,2,3-triazole) ginkgolide B (***5n**). Following the described procedure, 39.3 mg (66%) of compound **5n** were obtained from 46.2 mg (0.1 mmol) of 10-*O*-propargylated ginkgolide B (**3**). ^1^H-NMR (600 MHz, DMSO-*d_6_*) δ 8.46 (s, 1H), 7.52–7.47 (m, 2H), 7.46–7.41 (m, 2H), 6.47 (s, 1H), 6.20(s, 1H), 5.57 (d, *J* = 4.6 Hz, 1H), 5.45 (d, *J* = 12.2 Hz, 1H), 5.35–5.33 (m, 2H), 4.97 (d, *J* = 12.1 Hz, 1H), 4.64 (d, *J* = 7.2 Hz, 1H), 4.21 (dd, *J* = 7.2, 4.7 Hz, 1H), 2.89 (q, *J* = 7.0 Hz, 1H), 2.15 (s, 3H), 2.13 (dd, *J* = 13.7, 4.5 Hz, 1H), 1.91–1.80 (m, 1H), 1.75 (dd, *J* = 14.4 Hz, 4.4 Hz, 1H), 1.13 (d, *J* = 7.1 Hz, 3H), 1.03 (s, 9H). ^13^C-NMR (150 MHz, DMSO-*d_6_*) δ 176.83, 173.02, 170.63, 143.35, 136.56, 133.54, 131.90, 130.45, 127.53, 126.45, 125.28, 110.16, 99.33, 93.08, 82.94, 79.01, 75.62, 74.23, 72.43, 67.83, 63.57, 49.20, 41.90, 37.01, 32.22, 29.14 (3C), 17.84, 8.32. HRMS (ESI): *m*/*z* calcd for C_30_H_34_N_3_O_10_ [M + H]+: 596.2244, found 596.2249.

*10*-*O-(1-(2-Hydroxyphenyl)-1H-1,2,3-triazole) ginkgolide B* (**5o**). Following the described procedure, 22.1 mg (37%) of compound **5o** were obtained from 46.2 mg (0.1 mmol) of 10-*O*-propargylated ginkgolide B (3). ^1^H-NMR (600 MHz, DMSO-*d_6_*) δ 10.59 (s, 1H), 8.51 (s, 1H), 7.60 (dd, *J* = 7.9, 1.6 Hz, 1H), 7.36 (ddd, *J* = 8.2, 7.5, 1.7 Hz, 1H), 7.12 (dd, *J* = 8.2, 1.2 Hz, 1H), 7.00 (td, *J* = 7.8, 1.3 Hz, 1H), 6.46 (s, 1H), 6.20 (s, 1H), 5.64 (d, *J* = 4.7 Hz, 1H), 5.45 (d, *J* = 12.2 Hz, 1H), 5.34–5.32 (m, 2H), 4.93 (d, *J* = 12.1 Hz, 1H), 4.63 (d, *J* = 7.2 Hz, 1H), 4.22 (dd, *J* = 7.2, 4.5 Hz, 1H), 2.92–2.84 (m, 1H), 2.12 (dd, *J* = 13.3 Hz, 4.4 Hz, 1H), 1.84 (td, *J* = 13.8 Hz, 4.2 Hz, 1H), 1.74 (dd, *J* = 14.3 Hz, 4.3 Hz, 1H), 1.13 (d, *J* = 7.1 Hz, 3H), 1.02 (s, 9H). ^13^C-NMR (150 MHz, DMSO-*d_6_*) δ 175.74, 171.96, 169.55, 149.06, 141.97, 129.74, 124.60, 124.12, 123.73, 118.97, 116.45, 109.07, 98.23, 91.99, 81.83, 77.91, 74.66, 73.13, 71.35, 66.74, 62.61, 48.10, 40.98, 35.93, 31.15, 28.07 (3C), 7.23. HRMS (ESI): *m*/*z* calcd for C_29_H_32_N_3_O_11_ [M + H]^+^: 598.2037, found 598.2051.

*10*-*O-(1-(2-Cyanophenyl)-1H-1,2,3-triazole) ginkgolide B* (**5p**). Following the described procedure, 25.5 mg (42%) of compound **5p** were obtained from 46.2 mg (0.1 mmol) of 10-*O*-propargylated ginkgolide B (3). ^1^H-NMR (600 MHz, DMSO-*d_6_*) δ 8.74 (s, 1H), 8.16 (dd, *J* = 7.8, 1.3 Hz, 1H), 7.98 (td, *J* = 7.9, 1.4 Hz, 1H), 7.90–7.86 (m, 1H), 7.79 (td, *J* = 7.7, 1.1 Hz, 1H), 6.49 (s, 1H), 6.20 (s, 1H), 5.48 (d, *J* = 12.4 Hz, 2H), 5.37–5.30 (m, 2H), 4.99 (d, *J* = 12.3 Hz, 1H), 4.64 (d, *J* = 7.1 Hz, 1H), 4.22 (d, *J* = 7.1 Hz, 1H), 2.92–2.85 (m, 1H), 2.11 (dd, *J* = 13.5, 4.5 Hz, 1H), 1.86 (dt, *J* = 13.7, 7.0 Hz, 1H), 1.74 (dd, *J* = 14.4, 4.5 Hz, 1H), 1.13 (d, *J* = 7.1 Hz, 3H), 1.02 (s, 9H). ^13^C-NMR (150 MHz, DMSO-*d_6_*) δ 176.81, 172.97, 170.63, 144.21, 138.10, 135.41, 135.30, 130.92, 126.18, 124.91, 116.22, 110.16, 107.43, 99.41, 93.18, 82.97, 79.00, 75.76, 74.22, 72.41, 67.82, 63.48, 49.20, 42.04, 37.01, 32.22, 29.13 (3C), 8.35. HRMS (ESI): *m*/*z* calcd for C_30_H_31_N_4_O_10_ [M + H]^+^: 607.2040, found 607.2050.

*10*-*O-(1-(3-Methylphenyl)-1H-1,2,3-triazole) ginkgolide B* (**5q**). Following the described procedure, 26.8 mg (45%) of compound **5q** were obtained from 46.2 mg (0.1 mmol) of 10-*O*-propargylated ginkgolide B (3). ^1^H-NMR (600 MHz, DMSO-*d_6_*) δ 8.78 (s, 1H), 7.72 (t, *J* = 2.6 Hz, 1H), 7.68–7.63 (m, 1H), 7.50 (t, *J* = 7.8 Hz, 1H), 7.34 (d, *J* = 7.6 Hz, 1H), 6.47 (s, 1H), 6.20 (s, 1H), 5.50 (d, *J* = 4.5 Hz, 1H), 5.44 (d, *J* = 12.1 Hz, 1H), 5.33 (d, *J* = 4.8 Hz, 2H), 4.93 (d, *J* = 12.1 Hz, 1H), 4.64 (d, *J* = 7.1 Hz, 1H), 4.22–4.18 (m, 1H), 2.88 (q, *J* = 7.1 Hz, 1H), 2.42 (s, 3H), 2.11 (dd, *J* = 13.4 Hz, 4.4 Hz, 1H), 1.83 (td, *J* = 13.8 Hz, 4.2 Hz, 1H), 1.74 (dd, *J* = 14.4, 4.3 Hz, 1H), 1.13 (d, *J* = 7.1 Hz, 3H), 1.02 (s, 9H). ^13^C-NMR (150 MHz, DMSO-*d_6_*) δ 175.72, 171.93, 169.53, 143.06, 139.14, 135.83, 129.16, 128.93, 120.98, 120.04, 116.73, 109.08, 98.26, 92.00, 81.87, 77.89, 74.53, 73.16, 71.35, 66.72, 62.36, 48.11, 40.97, 35.93, 31.13, 28.04 (3C), 20.32, 7.25. HRMS (ESI): *m*/*z* calcd for C_30_H_34_N_3_O_10_ [M + H]^+^: 596.2244, found 596.2255.

*10*-*O-(1-(3-Isopropylphenyl)-1H-1,2,3-triazole) ginkgolide B* (**5r**). Following the described procedure, 34.3 mg (55%) of compound **5r** were obtained from 46.2 mg (0.1 mmol) of 10-*O*-propargylated ginkgolide B (3). ^1^H-NMR (600 MHz, DMSO-*d_6_*) δ 8.81 (s, 1H), 7.75 (t, *J* = 1.8 Hz, 1H), 7.68–7.67 (m, 1H), 7.53 (t, *J* = 7.9 Hz, 1H), 7.41 (d, *J* = 7.7 Hz, 1H), 6.50 (s, 1H), 6.20 (s, 1H), 5.54 (d, *J* = 4.6 Hz, 1H), 5.45 (d, *J* = 12.1 Hz, 1H), 5.34 (d, *J* = 3.4 Hz, 2H), 4.94 (d, *J* = 12.1 Hz, 1H), 4.64 (d, *J* = 7.1 Hz, 1H), 4.22 (dd, *J* = 7.1 Hz, 4.5 Hz, 1H), 3.06–2.99 (m, 1H), 2.89 (q, *J* = 7.0 Hz, 1H), 2.12 (dd, *J* = 13.4 Hz, 4.4 Hz, 1H), 1.83 (td, *J* = 13.8, 4.2 Hz, 1H), 1.74 (dd, *J* = 14.4 Hz, 4.3 Hz, 1H), 1.27 (d, *J* = 6.9 Hz, 6H), 1.13 (d, *J* = 7.1 Hz, 3H), 1.02 (s, 9H). ^13^C-NMR (150 MHz, DMSO-*d_6_*) δ 176.83, 173.02, 170.63, 150.14, 144.04, 136.99, 130.39, 127.50, 122.12, 118.67, 118.27, 110.16, 99.35, 93.06, 82.94, 78.99, 75.60, 74.25, 72.44, 67.81, 63.41, 49.19, 42.04, 37.00, 33.87, 32.20, 29.11 (3C), 24.11 (2C), 8.32. HRMS (ESI): *m*/*z* calcd for C_32_H_38_N_3_O_10_ [M + H]^+^: 624.2557, found 624.2565.

*10*-*O-(1-(3-Ethylcarboxyphenyl)-1H-1,2,3-triazole) ginkgolide B* (**5s**). Following the described procedure, 17.6 mg (27%) of compound **5s** were obtained from 46.2 mg (0.1 mmol) of 10-*O*-propargylated ginkgolide B (3). ^1^H-NMR (600 MHz, DMSO-*d_6_*) δ 8.94 (s, 1H), 8.40 (s, 1H), 8.18 (dd, *J* = 8.1, 0.8 Hz, 1H), 8.08 (dd, *J* = 7.8, 0.9 Hz, 1H), 7.79 (t, *J* = 8.0 Hz, 1H), 6.49 (s, 1H), 6.21 (s, 1H), 5.48 (d, *J* = 4.6 Hz, 1H), 5.46 (d, *J* = 12.2 Hz, 1H), 5.36–5.31 (m, 2H), 4.95 (d, *J* = 12.1 Hz, 1H), 4.64 (d, *J* = 7.1 Hz, 1H), 4.38 (q, *J* = 7.1 Hz, 2H), 4.22 (dd, *J* = 7.1, 4.6 Hz, 1H), 2.89 (q, *J* = 7.0 Hz, 1H), 2.11 (dd, *J* = 13.4 Hz, 4.4 Hz, 1H), 1.84 (td, *J* = 13.8 Hz, 4.2 Hz, 1H), 1.74 (dd, *J* = 14.4 Hz, 4.4 Hz, 1H), 1.36 (t, *J* = 7.1 Hz, 3H), 1.13 (d, *J* = 7.1 Hz, 3H), 1.02 (s, 9H). ^13^C-NMR (150 MHz, DMSO-*d_6_*) δ 176.81, 173.00, 170.61, 165.21, 144.41, 137.17, 132.08, 131.16, 129.77, 125.17, 122.46, 120.88, 110.16, 99.37, 93.11, 82.96, 78.98, 75.64, 74.23, 72.43, 67.80, 63.39, 61.85, 49.20, 42.04, 37.00, 32.21, 29.12 (3C), 14.59, 8.34. HRMS (ESI): *m*/*z* calcd for C_32_H_36_N_3_O_12_ [M + H]^+^: 654.2299, found 654.2309.

*10*-*O-(1-(3-Carboxyphenyl)-1H-1,2,3-triazole) ginkgolide B* (**5t**). Following the described procedure, 15.6 mg (25%) of compound **5t** were obtained from 46.2 mg (0.1 mmol) of 10-*O*-propargylated ginkgolide B (3). ^1^H-NMR (600 MHz, DMSO-*d_6_*) δ 13.45 (s, 1H), 8.94 (s, 1H), 8.38 (s, 1H), 8.16–8.14 (m, 1H), 8.11–7.99 (m, 1H), 7.76 (t, *J* = 7.9 Hz, 1H), 6.47 (s, 1H), 6.21 (s, 1H), 5.47–5.44 (m, 2H), 5.35–5.28 (m, 2H), 4.94 (d, *J* = 12.1 Hz, 1H), 4.64 (d, *J* = 7.1 Hz, 1H), 4.21 (dd, *J* = 7.1 Hz, 4.6 Hz, 1H), 2.89 (q, *J* = 7.1 Hz, 1H), 2.11 (dd, *J* = 13.4 Hz, 4.4 Hz, 1H), 1.84 (td, *J* = 13.8 Hz, 4.2 Hz, 1H), 1.74 (dd, *J* = 14.4 Hz, 4.4 Hz, 1H), 1.13 (d, *J* = 7.1 Hz, 3H), 1.02 (s, 9H). ^13^C-NMR (150 MHz, DMSO-*d_6_*) δ 176.81, 173.01, 170.62, 166.75, 144.21, 136.99, 133.01, 130.95, 129.88, 124.72, 122.30, 120.85, 110.07, 99.24, 92.99, 82.94, 78.93, 75.54, 74.21, 72.38, 67.74, 63.34, 49.20, 42.04, 36.74, 32.20, 29.12 (3C), 8.26. HRMS (ESI): *m*/*z* calcd for C_30_H_32_N_3_O_12_ [M + H]^+^: 626.1986, found 626.1998.

*10*-*O-(1-(4-Methylphenyl)-1H-1,2,3-triazole) ginkgolide B* (**5u**). Following the described procedure, 31.5 mg (53%) of compound **5u** were obtained from 46.2 mg (0.1 mmol) of 10-*O*-propargylated ginkgolide B (3). ^1^H-NMR (600 MHz, DMSO-*d_6_*) δ 8.75 (s, 1H), 7.76 (d, *J* = 8.4 Hz, 2H), 7.42 (d, *J* = 8.1 Hz, 2H), 6.46 (s, 1H), 6.20 (s, 1H), 5.53 (d, *J* = 4.6 Hz, 1H), 5.44 (d, *J* = 12.2 Hz, 1H), 5.33 (d, *J* = 3.2 Hz, 2H), 4.92 (d, *J* = 12.1 Hz, 1H), 4.63 (d, *J* = 7.1 Hz, 1H), 4.21 (dd, *J* = 7.2, 4.6 Hz, 1H), 2.88 (q, *J* = 7.0 Hz, 1H), 2.39 (s, 3H), 2.11 (dd, *J* = 13.4 Hz, 4.4 Hz, 1H), 1.83 (td, *J* = 13.8 Hz, 4.2 Hz, 1H), 1.73 (dd, *J* = 14.4 Hz, 4.3 Hz, 1H), 1.13 (d, *J* = 7.1 Hz, 3H), 1.02 (s, 9H). ^13^C-NMR (150 MHz, DMSO-*d_6_*) δ 175.72, 171.94, 169.52, 142.94, 137.81, 133.34, 129.57 (2C), 120.81, 119.40 (2C), 109.01, 98.21, 91.97, 81.84, 77.84, 74.37, 73.03, 71.26, 66.65, 62.28, 47.99, 40.81, 35.83, 30.92, 28.04 (3C), 19.99, 7.01. HRMS (ESI): *m*/*z* calcd for C_30_H_34_N_3_O_10_ [M + H]^+^: 596.2244, found 596.2251.

*10*-*O-(1-(4-Methoxyphenyl)-1H-1,2,3-triazole) ginkgolide B* (**5v**). Following the described procedure, 20.8 mg (34%) of compound **5v** were obtained from 46.2 mg (0.1 mmol) of 10-*O*-propargylated ginkgolide B (3). ^1^H-NMR (600 MHz, DMSO-*d_6_*) δ 8.69 (s, 1H), 7.78 (d, *J* = 9.0 Hz, 2H), 7.15 (d, *J* = 9.1 Hz, 2H), 6.47 (s, 1H), 6.20 (s, 1H), 5.57 (d, *J* = 4.6 Hz, 1H), 5.44 (d, *J* = 12.1 Hz, 1H), 5.33 (s, 2H), 4.92 (d, *J* = 12.1 Hz, 1H), 4.63 (d, *J* = 7.2 Hz, 1H), 4.21 (dd, *J* = 7.2, 4.6 Hz, 1H), 3.84 (s, 3H), 2.88 (q, *J* = 7.1 Hz, 1H), 2.11 (dd, *J* = 13.4 Hz, 4.4 Hz, 1H), 1.83 (td, *J* = 13.8 Hz, 4.2 Hz, 1H), 1.74 (dd, *J* = 14.4 Hz, 4.3 Hz, 1H), 1.13 (d, *J* = 7.1 Hz, 3H), 1.02 (s, 9H). ^13^C-NMR (150 MHz, DMSO-*d_6_*) δ 176.83, 173.02, 170.63, 159.92, 144.00, 130.31, 122.40 (2C), 121.94, 115.42 (2C), 110.16, 99.34, 93.08, 82.93, 79.00, 75.43, 74.13, 72.34, 67.75, 63.27, 55.84, 49.09, 41.96, 36.94, 32.01, 28.95 (3C), 8.19. HRMS (ESI): *m*/*z* calcd for C_30_H_34_N_3_O_11_ [M + H]^+^: 612.2193, found 612.2205.

*10*-*O-(1-(4-Fluorophenyl)-1H-1,2,3-triazole) ginkgolide B* (**5w**). Following the described procedure, 13.5 mg (22%) of compound **5w** were obtained from 46.2 mg (0.1 mmol) of 10-*O*-propargylated ginkgolide B (3). ^1^H-NMR (600 MHz, DMSO-*d_6_*) δ 8.69 (s, 1H), 7.93–7.89 (m, 2H), 7.49–7.44 (m, 2H), 6.64 (s, 1H), 6.19 (s, 1H), 5.63 (d, *J* = 4.5 Hz, 1H), 5.46 (d, *J* = 12.2 Hz, 1H), 5.37 (d, *J* = 4.1 Hz, 1H), 5.31 (s, 1H), 4.97 (d, *J* = 12.2 Hz, 1H), 4.65 (d, *J* = 7.1 Hz, 1H), 4.24 (dd, *J* = 7.1 Hz, 4.4 Hz, 1H), 2.91 (q, *J* = 7.1 Hz, 1H), 2.14 (dd, *J* = 13.5 Hz, 4.5 Hz, 1H), 1.86 (td, *J* = 13.9 Hz, 4.2 Hz, 1H), 1.75 (dd, *J* = 14.4 Hz, 4.5 Hz, 1H), 1.15 (d, *J* = 7.1 Hz, 3H), 1.03 (s, 9H). ^13^C-NMR (150 MHz, DMSO-*d_6_*) δ 177.01, 172.98, 170.81, 162.28 (d, *J*_C-F_ = 246.4 Hz), 144.27, 133.33 (d, *J*_C-F_ = 2.5 Hz), 123.18 (d, *J*_C-F_ = 8.9 Hz, 2C), 122.14, 117.30 (d, *J*_C-F_ = 23.3 Hz, 2C), 110.18, 99.41, 93.08, 82.95, 79.16, 75.63, 74.28, 72.44, 67.85, 63.41, 49.21, 42.02, 36.95, 32.11, 29.03 (3C), 7.94. HRMS (ESI): *m*/*z* calcd for C_29_H_31_FN_3_O_10_ [M + H]^+^: 600.1993, found 600.2010.

*10*-*O-(1-(4-Bromophenyl)-1H-1,2,3-triazole) ginkgolide B* (**5x**). Following the described procedure, 18.5 mg (28%) of compound **5x** were obtained from 46.2 mg (0.1 mmol) of 10-*O*-propargylated ginkgolide B (3). ^1^H-NMR (600 MHz, DMSO-*d_6_*) δ 8.83 (s, 1H), 7.88–7.85 (m, 2H), 7.85–7.81 (m, 2H), 6.47 (s, 1H), 6.20 (s, 1H), 5.47 (d, *J* = 4.6 Hz, 1H), 5.45 (d, *J* = 12.2 Hz, 1H), 5.33 (d, *J* = 4.1 Hz, 1H), 4.93 (d, *J* = 12.1 Hz, 1H), 4.63 (d, *J* = 7.1 Hz, 1H), 4.21 (dd, *J* = 7.1 Hz, 4.6 Hz, 1H), 2.88 (q, *J* = 7.1 Hz, 1H), 2.11 (dd, *J* = 13.4 Hz, 4.4 Hz, 1H), 1.83 (td, *J* = 13.8 Hz, 4.2 Hz, 1H), 1.74 (dd, *J* = 14.4 Hz, 4.3 Hz, 1H), 1.13 (d, *J* = 7.1 Hz, 3H), 1.02 (s, 9H). ^13^C-NMR (150 MHz, DMSO-*d_6_*) δ 176.80, 172.99, 170.60, 144.39, 136.14, 133.35 (2C), 122.63 (2C), 122.20, 122.09, 110.16, 99.36, 93.10, 82.96, 78.97, 75.65, 74.23, 72.42, 67.79, 63.33, 49.02, 41.91, 36.81, 32.10, 28.98 (3C), 8.26. HRMS (ESI): *m*/*z* calcd for C_29_H_31_BrN_3_O_10_ [M + H]^+^: 660.1193, found 660.1208.

*10*-*O-(1-(4-Trifluoromethylphenyl)-1H-1,2,3-triazole)ginkgolide B* (**5y**). Following the described procedure, 20.1 mg (31%) of compound **5y** were obtained from 46.2 mg (0.1 mmol) of 10-*O*-propargylated ginkgolide B (3). ^1^H-NMR (600 MHz, DMSO-*d_6_*) δ 8.95 (s, 1H), 8.15 (d, *J* = 8.4 Hz, 2H), 8.02 (d, *J* = 8.6 Hz, 2H), 6.47 (s, 1H), 6.21 (s, 1H), 5.47 (d, *J* = 12.2 Hz, 1H), 5.45 (d, *J* = 4.6 Hz, 1H), 5.35–5.32 (m, 2H), 4.95 (d, *J* = 12.1 Hz, 1H), 4.64 (d, *J* = 7.1 Hz, 1H), 4.21 (dd, *J* = 7.1 Hz, 4.6 Hz, 1H), 2.89 (q, *J* = 7.1 Hz, 1H), 2.11 (dd, *J* = 13.4 Hz, 4.4 Hz, 1H), 1.84 (td, *J* = 13.8 Hz, 4.2 Hz, 1H), 1.74 (dd, *J* = 14.4 Hz, 4.4 Hz, 1H), 1.13 (d, *J* = 7.1 Hz, 3H), 1.02 (s, 9H). ^13^C-NMR (150 MHz, DMSO-*d_6_*) δ 176.81, 172.98, 170.61, 144.59, 139.72, 129.34 (q, *J*_C-F_ = 32.1 Hz), 127.78 (q, *J*_C-F_ = 3.6 Hz, 2C), 124.27 (q, *J* = 270.5 Hz), 122.50, 121.21 (2C), 110.16, 99.38, 93.12, 82.97, 78.97, 75.68, 74.24, 72.42, 67.80, 63.39, 49.19, 42.04, 37.01, 32.21, 29.12 (3C), 8.34. HRMS (ESI): *m*/*z* calcd for C_30_H_31_F_3_N_3_O_10_ [M + H]^+^: 650.1962, found 650.1967.

*10*-*O-(1-(4-Nitrophenyl)-1H-1,2,3-triazole) ginkgolide B* (**5z**). Following the described procedure, 21.9 mg (35%) of compound **5z** were obtained from 46.2 mg (0.1 mmol) of 10-*O*-propargylated ginkgolide B (3). ^1^H-NMR (600 MHz, DMSO-*d_6_*) δ 9.00 (s, 1H), 8.49–8.47 (m, 2H), 8.23–8.20 (m, 2H), 6.47 (s, 1H), 6.20 (d, *J* = 6.2 Hz, 1H), 5.46 (t, *J* = 12.2 Hz, 1H), 5.40 (d, *J* = 4.6 Hz, 1H), 5.36–5.32 (m, 2H), 4.95 (d, *J* = 12.1 Hz, 1H), 4.64 (d, *J* = 7.1 Hz, 1H), 4.21 (dd, *J* = 7.1 Hz, 4.6 Hz, 1H), 2.93–2.84 (m, 1H), 2.11 (dd, *J* = 13.4 Hz, 4.4 Hz, 1H), 1.84 (td, *J* = 13.8 Hz, 4.2 Hz, 1H), 1.74 (dd, *J* = 14.4 Hz, 4.4 Hz, 1H), 1.13 (d, *J* = 7.1 Hz, 3H), 1.02 (s, 9H). ^13^C-NMR (150 MHz, DMSO-*d_6_*) δ 176.79, 172.97, 170.60, 147.38, 144.82, 141.16, 126.13 (2C), 122.76, 121.30 (2C), 110.16, 99.39, 93.14, 82.98, 78.96, 75.74, 74.23, 72.41, 67.79, 63.37, 49.19, 42.04, 37.01, 32.22, 29.13 (3C), 8.35. HRMS (ESI): *m*/*z* calcd for C_29_H_31_N_4_O_12_ [M + H]^+^: 627.1938, found 627.1948.

*10-O-(1-(4-Methylcarboxyphenyl)-1H-1,2,3-triazole) ginkgolide B* (**5aa**). Following the described procedure, 18.5 mg (29%) of compound **5aa** were obtained from 46.2 mg (0.1 mmol) of 10-*O*-propargylated ginkgolide B (3), ^1^H-NMR (600 MHz, DMSO-*d_6_*) δ 8.94 (s, 1H), 8.20–8.16 (m, 2H), 8.09–8.06 (m, 2H), 6.47 (s, 1H), 6.21 (s, 1H), 5.46 (d, *J* = 12.2 Hz, 1H), 5.42 (d, *J* = 4.6 Hz, 1H), 5.36–5.28 (m, 2H), 4.94 (d, *J* = 12.1 Hz, 1H), 4.64 (d, *J* = 7.1 Hz, 1H), 4.21 (dd, *J* = 7.1 Hz, 4.6 Hz, 1H), 3.90 (s, 3H), 2.88 (q, *J* = 7.1 Hz, 1H), 2.11 (dd, *J* = 13.4 Hz, 4.4 Hz, 1H), 1.84 (td, *J* = 13.8 Hz, 4.2 Hz, 1H), 1.74 (dd, *J* = 14.4 Hz, 4.4 Hz, 1H), 1.13 (d, *J* = 7.1 Hz, 3H), 1.02 (s, 9H). ^13^C-NMR (150 MHz, DMSO-*d_6_*) δ 176.80, 172.98, 170.60, 165.79, 144.58, 140.11, 131.53 (2C), 130.08, 122.40, 120.57 (2C), 110.16, 99.38, 93.12, 82.97, 78.97, 75.71, 74.23, 72.42, 67.79, 63.41, 52.64, 49.03, 42.04, 36.70, 32.12, 28.90 (3C), 8.29. HRMS (ESI): *m*/*z* calcd for C_31_H_34_N_3_O_12_ [M + H]^+^: 640.2142, found 640.2154.

*10*-*O-(1-(4-Carboxyphenyl)-1H-1,2,3-triazole) ginkgolide B* (**5bb**). Following the described procedure, 16.3 mg (26%) of compound **5bb** were obtained from 46.2 mg (0.1 mmol) of 10-*O*-propargylated ginkgolide B (3). ^1^H-NMR (600 MHz, DMSO-*d_6_*) δ 8.88 (s, 1H), 8.16 (d, *J* = 8.4 Hz, 2H), 8.16 (d, *J* = 8.4 Hz, 2H), 6.50 (s, 1H), 6.19 (s, 1H), 5.49–5.44 (m, 2H), 5.35–5.32 (m, 2H), 4.94 (d, *J* = 12.0 Hz, 1H), 4.63 (d, *J* = 7.2 Hz, 1H), 4.21 (dd, *J* = 7.2 Hz, 4.2 Hz, 1H), 2.88 (q, *J* = 7.2 Hz, 1H), 2.11 (dd, *J* = 13.4 Hz, 4.4 Hz, 1H), 1.83 (td, *J* = 13.8 Hz, 4.2 Hz, 1H), 1.73 (dd, *J* = 14.4 Hz, 4.4 Hz, 1H), 1.13 (d, *J* = 7.1 Hz, 3H), 1.02 (s, 9H). ^13^C-NMR (150 MHz, DMSO-*d_6_*) δ 176.84, 172.98, 170.50, 166.86, 144.40, 139.78, 131.60 (2C), 131.29, 122.20, 120.28 (2C), 109.95, 99.28, 93.09, 82.95, 79.01, 75.55, 74.25, 72.32, 67.76, 63.29, 49.16, 41.92, 36.87, 32.10, 29.11 (3C), 8.25. HRMS (ESI): *m*/*z* calcd for C_30_H_32_N_3_O_12_ [M + H]^+^: 626.1986, found 626.1998.

*10*-*O-(1-(3,5-Dimethoxyphenyl)-1H-1,2,3-triazole) ginkgolide B* (**5cc**). Following the described procedure, 22.5 mg (35%) of compound **5cc** were obtained from 46.2 mg (0.1 mmol) of 10-*O*-propargylated ginkgolide B (3). ^1^H-NMR (600 MHz, DMSO-*d_6_*) δ 8.79 (s, 1H), 7.05 (d, *J* = 2.4 Hz, 2H), 6.63 (t, *J* = 2.4 Hz, 1H), 6.52 (s, 1H), 6.18 (s, 1H), 5.52 (s, *J* = 4.2 Hz, 1H), 5.43 (d, *J* = 12.0 Hz, 1H), 5.34 (d, *J* = 4.2 Hz, 1H), 5.32 (s, 1H), 4.92 (d, *J* = 12.0 Hz, 1H), 4.63 (d, *J* = 7.2 Hz, 1H), 4.22 (dd, *J* = 7.2 Hz, 4.2 Hz, 1H), 3.84 (s, 6H), 2.88 (q, *J* = 7.2 Hz, 1H), 2.11 (dd, *J* = 13.4 Hz, 4.4 Hz, 1H), 1.83 (td, *J* = 13.8, 4.2 Hz, 1H), 1.73 (dd, *J* = 14.4 Hz, 4.4 Hz, 1H), 1.13 (d, *J* = 7.1 Hz, 3H), 1.02 (s, 9H). ^13^C-NMR (150 MHz, DMSO-*d_6_*) δ 176.87, 173.00, 170.66, 161.70 (2C), 144.08, 138.40, 122.15, 110.17, 100.86, 99.37, 98.98 (2C), 93.05, 82.94, 79.03, 75.60, 74.26, 72.43, 67.81, 63.37, 56.21 (2C), 49.20, 42.03, 36.98, 32.17, 29.09 (3C), 8.31. HRMS (ESI): *m*/*z* calcd for C_31_H_36_N_3_O_12_ [M + H]^+^: 642.2299, found 642.2308.

*10*-*O-(1-(3-Chloro-4-fluorophenyl)-1H-1,2,3-triazole) ginkgolide B* (**5dd**). Following the described procedure, 29.2 mg (46%) of compound **5dd** were obtained from 46.2 mg (0.1 mmol) of 10-*O*-propargylated ginkgolide B (3). ^1^H-NMR (600 MHz, DMSO-*d_6_*) δ 8.83 (s, 1H), 8.20–8.18 (m, 1H), 8.00–7.88 (m, 1H), 7.75–7.60 (m, 1H), 6.48 (s, 1H), 6.20 (s, 1H), 5.46–5.41 (m, 2H), 5.33 (s, 2H), 4.93 (d, *J* = 12.0 Hz, 1H), 4.64 (d, *J* = 7.1 Hz, 1H), 4.23–4.17 (m, 1H), 2.88 (q, *J* = 7.2 Hz, 1H), 2.11 (dd, *J* = 13.4 Hz, 4.4 Hz, 1H), 1.83 (td, *J* = 13.8 Hz, 4.2 Hz, 1H), 1.73 (dd, *J* = 14.4 Hz, 4.4 Hz, 1H), 1.13 (d, *J* = 7.1 Hz, 3H), 1.02 (s, 9H). HRMS (ESI): *m*/*z* calcd for C_29_H_30_ClFN_3_O_10_ [M + H]^+^: 634.1604, found 634.1619.

*10-O-(1-(3-Pyridinyl)-1H-1,2,3-triazole) ginkgolide B* (**5ee**). Following the described procedure, 45.7 mg (67%) of compound **5ee** were obtained from 46.2 mg (0.1 mmol) of 10-*O*-propargylated ginkgolide B (3). ^1^H-NMR (600 MHz, DMSO-*d_6_*) δ 9.13 (d, *J* = 2.5 Hz, 1H), 8.88 (s, 1H), 8.71 (d, *J* = 4.8 Hz, 1H), 8.35–8.30 (m, 1H), 7.68 (dd, *J* = 8.4, 4.8 Hz, 1H), 6.49 (s, 1H), 6.21 (s, 1H), 5.49 (d, *J* = 4.8 Hz, 1H), 5.47 (d, *J* = 12.0 Hz, 1H), 5.34 (d, *J* = 3.2 Hz, 2H), 4.95 (d, *J* = 12.1 Hz, 1H), 4.64 (d, *J* = 7.1 Hz, 1H), 4.21 (dd, *J* = 7.1 Hz, 4.5 Hz, 1H), 2.88 (q, *J* = 7.0 Hz, 1H), 2.12 (dd, *J* = 13.5 Hz, 4.5 Hz, 1H), 1.85 (td, *J* = 13.8 Hz, 4.2 Hz, 1H), 1.74 (dd, *J* = 14.4 Hz, 4.4 Hz, 1H), 1.13 (d, *J* = 7.1 Hz, 3H), 1.03 (s, 9H). ^13^C-NMR (150 MHz, DMSO-*d_6_*) δ 176.80, 172.99, 170.59, 150.41, 144.45, 141.84, 133.59, 128.59, 125.10, 122.47, 110.07, 99.32, 92.92, 82.84, 78.99, 75.45, 73.96, 72.07, 67.61, 63.29, 48.94, 42.04, 36.71, 31.75, 29.12 (3C), 8.16. HRMS (ESI): *m*/*z* calcd for C_28_H_31_N_4_O_10_ [M + H]^+^: 583.2040, found 583.2056.

*10*-*O-(1-Benzyl-1H-1,2,3-triazole) ginkgolide B* (**5ff**). Following the described procedure, 42.8 mg (72%) of compound **5ff** were obtained from 46.2 mg (0.1 mmol) of 10-*O*-propargylated ginkgolide B (3). ^1^H-NMR (600 MHz, DMSO-*d_6_*) δ 8.17 (s, 1H), 7.41–7.30 (m, 5H), 6.47 (s, 1H), 6.17 (s, 1H), 5.64 (d, *J* = 4.4 Hz, 1H), 5.61 (s, 2H), 5.35–5.31 (m, 2H), 5.27 (s, 1H), 4.84 (d, *J* = 12.1 Hz, 1H), 4.62 (d, *J* = 7.2 Hz, 1H), 4.17 (dd, *J* = 7.1 Hz, 4.5 Hz, 1H), 2.86 (q, *J* = 7.0 Hz, 1H), 2.09 (dd, *J* = 12.9 Hz, 3.9 Hz, 1H), 1.77 (td, *J* = 13.8 Hz, 4.2 Hz, 1H), 1.70 (dd, *J* = 14.4 Hz, 4.4 Hz, 1H), 1.12 (d, *J* = 7.1 Hz, 3H), 0.98 (s, 9H). ^13^C-NMR (150 MHz, DMSO-*d_6_*) δ 176.83, 172.99, 170.62, 143.50, 136.17, 129.26 (2C), 128.69, 128.48 (2C), 123.01, 109.77, 98.98, 92.78, 82.56, 78.85, 75.51, 74.03, 72.28, 67.54, 63.16, 53.19, 48.92, 41.92, 36.77, 31.99, 28.81 (3C), 8.15. HRMS (ESI): *m*/*z* calcd for C_30_H_34_N_3_O_10_ [M + H]^+^: 596.2244, found 596.2266.

*10*-*O-(((Anthracen-2-yloxy)methyl)-1H-1,2,3-triazole) ginkgolide B* (**5gg**). Following the described procedure, 43.5 mg (70%) of compound **5gg** were obtained from 46.2 mg (0.1 mmol) of 10-*O*-propargylated ginkgolide B (3). ^1^H-NMR (600 MHz, DMSO-*d_6_*) δ 8.22 (s, 1H), 7.93 (dd, *J* = 7.8 Hz, 1.3 Hz, 1H), 7.73 (td, *J* = 7.7 Hz, 1.4 Hz, 1H), 7.58 (td, *J* = 7.7 Hz, 1.2 Hz, 1H), 7.38 (dd, *J* = 7.9 Hz, 1.1 Hz, 1H), 6.45 (s, 1H), 6.17 (s, 1H), 5.83 (s, 2H), 5.58 (d, *J* = 4.6 Hz, 1H), 5.36 (d, *J* = 12.2 Hz, 1H), 5.31 (d, *J* = 3.9 Hz, 1H), 5.28 (s, 1H), 4.86 (d, *J* = 12.1 Hz, 1H), 4.61 (d, *J* = 7.2 Hz, 1H), 4.17 (dd, *J* = 7.2 Hz, 4.2 Hz, 1H), 4.03 (q, *J* = 7.1 Hz, 1H), 2.87 (q, *J* = 7.1 Hz, 1H), 2.50 (s, 2H), 2.09 (dd, *J* = 13.0 Hz, 4.1 Hz, 1H), 1.99 (s, 2H), 1.83–1.68 (m, 2H), 1.18 (t, *J* = 7.1 Hz, 2H), 1.12 (d, *J* = 7.1 Hz, 3H), 0.98 (s, 9H). ^13^C NMR (150 MHz, DMSO-*d_6_*) δ 176.91, 172.97, 170.70, 143.52, 138.80, 134.36, 133.90, 130.22, 129.89, 124.13, 117.45, 111.79, 110.15, 99.32, 82.87, 79.06, 75.62, 74.19, 72.42, 67.81, 63.54, 60.34, 51.71, 49.15, 42.02, 36.92, 32.12, 29.04(3C), 8.25. HRMS (ESI): *m*/*z* calcd for C_31_H_32_N_4_O_10_ [M + H]^+^: 621.2198, found 621.2170.

*10-O-(1-Phenyl-1H-1,2,3-triazole) ginkgolide A* (**5′a**). Following the described procedure, 36.2 mg (64%) of compound **5′a** were obtained from 44.6 mg (0.1 mmol) of 10-*O*-propargylated ginkgolide A (**3′**). ^1^H-NMR (600 MHz, DMSO-*d_6_*) δ 8.51 (s, 1H), 7.48 (s, 2H), 7.43 (d, *J* = 16.7 Hz, 2H), 6.45–6.32 (m, 1H), 6.15 (s, 1H), 5.36 (d, *J* = 12.0 Hz, 1H), 5.23 (s, 1H), 4.95 (d, *J* = 4.0 Hz, 1H), 4.89 (d, *J* = 11.9 Hz, 1H), 4.83 (dd, *J* = 8.4 Hz, 7.3 Hz, 1H), 3.85–3.71 (m, 2H), 3.62–3.56 (m, 1H), 3.17 (d, *J* = 5.3 Hz, 1H), 2.95 (q, *J* = 7.1 Hz, 1H), 2.75 (dd, *J* = 15.1 Hz, 7.2 Hz, 1H), 2.04 (dd, *J* = 13.6 Hz, 5.0 Hz, 1H), 2.01–1.91 (m, 2H), 1.91–1.80 (m, 2H), 1.79–1.70 (m, 3H), 1.70–1.63 (m, 1H), 1.13 (d, *J* = 7.1 Hz, 3H), 1.02 (s, 9H). ^13^C-NMR (150 MHz, DMSO-*d_6_*) δ 176.98, 173.22, 171.05, 144.45, 130.41(2C), 122.91, 120.67(2C), 110.14, 100.81, 87.96, 86.50, 85.31, 75.74, 68.63, 66.81, 66.19, 63.35, 49.16, 36.78, 36.45, 33.62, 32.19, 29.17(3C), 23.75, 8.59. HRMS (ESI): *m*/*z* calcd for C_29_H_31_N_3_O_9_ [M + H]^+^: 566.2133, found 566.2111.

*10*-*O-(1-(2-Methylphenyl)-1H-1,2,3-triazole) ginkgolide A* (**5′n**). Following the described procedure, 40.0 mg (69%) of compound **5′n** were obtained from 44.6 mg (0.1 mmol) of 10-*O*-propargylated ginkgolide A (**3′**). ^1^H-NMR (600 MHz, DMSO-*d_6_*) δ 8.51 (s, 1H), 7.52–7.48 (m, 2H), 7.44 (dd, *J* = 2.2, 1.0 Hz, 2H), 6.40 (s, 1H), 6.15 (s, 1H), 5.40–5.33 (m, 2H), 5.23 (s, 1H), 4.95 (d, *J* = 4.1 Hz, 1H), 4.89 (d, *J* = 11.9 Hz, 1H), 4.83 (d, *J* = 1.1 Hz, 1H), 3.82–3.75 (m, 2H), 3.60 (s, 1H), 3.17 (d, *J* = 5.3 Hz, 1H), 2.95 (d, *J* = 7.2 Hz, 1H), 2.75 (dd, *J* = 15.1 Hz, 7.2 Hz, 1H), 2.15 (s, 3H), 1.97 (s, 1H), 1.78–1.69 (m, 3H), 1.13 (d, *J* = 7.1 Hz, 3H), 1.02 (s, 9H). ^13^C-NMR (150 MHz, DMSO-*d_6_*) δ 176.97, 173.19, 171.05, 143.65, 136.71, 133.55, 131.87, 130.34, 127.50, 126.51, 126.03, 110.13, 100.79, 97.64, 87.94, 86.50, 85.35, 75.74, 67.49, 63.53, 49.07, 41.66, 40.47, 36.78, 32.20, 29.16(3C), 17.87, 8.59. HRMS (ESI): *m*/*z* calcd for C_30_H_33_N_3_O_9_ [M + H] ^+^: 580.2197, found 580.2266.

*10*-*O-(1-(2-Cyanophenyl)-1H-1,2,3-triazole) ginkgolide* A (**5′p**)., Following the described procedure, 34.3 mg (58%) of compound **5′p** were obtained from 446 mg (0.1 mmol) of 10-*O*-propargylated ginkgolide A (**3′**). ^1^H-NMR (600 MHz, DMSO-*d_6_*) δ 8.82 (s, 1H), 8.16 (dd, *J* = 7.9, 1.5 Hz, 1H), 7.98 (td, *J* = 7.9, 1.5 Hz, 1H), 7.91 (dd, *J* = 8.2, 1.2 Hz, 1H), 7.79 (td, *J* = 7.7, 1.2 Hz, 1H), 6.41 (s, 1H), 6.16 (s, 1H), 5.41 (d, *J* = 11.9 Hz, 1H), 5.39–5.35 (m, 2H), 5.26 (s, 1H), 4.97–4.87 (m, 2H), 4.82 (t, *J* = 7.8 Hz, 1H), 2.95 (q, *J* = 7.1 Hz, 1H), 2.79–2.72 (m, 1H), 1.40 (s, 3H), 1.13 (d, *J* = 7.2 Hz, 3H), 1.04 (s, 9H). ^13^C-NMR (150 MHz, DMSO-*d_6_*) δ 174.85, 171.03, 168.75, 142.36, 133.25, 133.10, 128.73, 128.69, 124.10, 123.44, 110.75, 108.04, 108.01, 98.77, 95.53, 85.83, 84.40, 83.30, 73.77, 66.57, 64.74, 61.33, 47.05, 39.68, 34.68, 34.37, 31.51(3C), 8.07. HRMS (ESI): *m*/*z* calcd for C_30_H_30_N_4_O_9_ [M + H] ^+^: 591.2093, found 591.2062.

*10*-*O-(1-Benzyl -1H-1,2,3-triazole) ginkgolide A* (**5′ff**). Following the described procedure, 39.4 mg (68%) of compound **5′ff** were obtained from 44.6 mg (0.1 mmol) of 10-*O*-propargylated ginkgolide A (**3′**). ^1^H-NMR (600 MHz, DMSO-*d_6_*) δ 8.25 (s, 1H), 7.38 (dd, *J* = 8.0, 6.4 Hz, 2H), 7.34–7.22 (m, 3H), 6.40 (s, 1H), 6.12 (s, 1H), 5.61 (d, *J* = 6.9 Hz, 2H), 5.24 (d, *J* = 11.6 Hz, 1H), 5.16 (s, 1H), 4.92 (d, *J* = 4.0 Hz, 1H), 4.82 (t, *J* = 7.8 Hz, 1H), 4.76 (d, *J* = 11.6 Hz, 1H), 4.03 (q, *J* = 7.1 Hz, 1H), 2.94 (d, *J* = 7.2 Hz, 1H), 2.65 (dd, *J* = 15.1 Hz, 7.2 Hz, 1H), 2.50 (p, *J* = 1.8 Hz, 3H), 2.05–1.99 (m, 1H), 1.99 (s, 1H), 1.90 (d, *J* = 4.4 Hz, 1H), 1.84 (dd, *J* = 15.1, 8.4 Hz, 1H), 1.70 (dd, *J* = 14.1, 4.7 Hz, 1H), 1.17 (t, *J* = 7.1 Hz, 1H), 1.12 (dd, *J* = 7.1, 4.0 Hz, 3H), 0.98 (d, *J* = 3.6 Hz, 9H). ^13^C-NMR (150 MHz, DMSO-*d_6_*) δ 177.20, 173.13, 171.32, 136.27, 129.28(2C), 128.74, 128.39, 124.82, 110.16, 110.03, 101.11, 88.10, 86.53, 85.77, 75.61, 68.76, 66.93, 63.50, 63.40, 53.55, 53.35, 49.15, 40.86, 36.82, 32.04, 29.02(3C), 8.71. HRMS (ESI): *m*/*z* calcd for C_30_H_33_N_3_O_9_ [M + H] ^+^: 580.2297, found 580.2267.

*10-O-(((Anthracen-2-yloxy)methyl)-1H-1,2,3-triazole) ginkgolide A* (**5′gg**). Following the described procedure, 36.9 mg (61%) of compound **5′gg** were obtained from 44.6 mg (0.1 mmol) of 10-*O*-propargylated ginkgolide A (**3′**). ^1^H-NMR (600 MHz, DMSO-*d_6_*) δ 8.29 (s, 1H), 7.92 (dd, *J* = 7.7, 1.4 Hz, 1H), 7.82–7.69 (m, 1H), 7.57 (td, *J* = 7.7, 1.2 Hz, 1H), 7.44–7.26 (m, 1H), 6.40 (s, 1H), 6.13 (s, 1H), 5.83 (s, 2H), 5.27 (d, *J* = 11.8 Hz, 1H), 5.18 (s, 1H), 4.90 (d, *J* = 4.0 Hz, 1H), 4.83 (dd, *J* = 8.2 Hz, 7.3 Hz, 1H), 4.78 (d, *J* = 11.7 Hz, 1H), 4.03 (q, *J* = 7.1 Hz, 19H), 2.95 (q, *J* = 7.2 Hz, 1H), 2.66 (dd, *J* = 15.2 Hz, 7.2 Hz, 1H), 2.51 (p, *J* = 1.8 Hz, 4H), 1.90–1.82 (m, 2H), 1.72 (dd, *J* = 14.1 Hz, 4.7 Hz, 2H), 1.40 (s, 3H), 1.13 (d, *J* = 7.2 Hz, 3H), 0.99 (d, *J* = 5.3 Hz, 9H). ^13^C-NMR (150 MHz, DMSO-*d_6_*) δ 176.93, 173.14, 170.81, 143.66, 139.26, 134.29, 133.85, 129.91, 129.68, 125.44, 117.45, 111.68, 110.09, 100.82, 87.90, 86.50, 85.33, 75.74, 68.63, 66.81, 63.47, 60.22, 51.47, 49.03, 40.89, 36.40, 32.14, 29.10(3C), 8.56. HRMS (ESI): *m*/*z* calcd for C_31_H_32_N_4_O_9_ [M + H] ^+^: 605.2249, found 605.2219.

*10*-*O-(1-Phenyl-1H-1,2,3-triazole) ginkgolide C* (**5″a**). Following the described procedure, 31.1 mg (52%) of compound **5″a** were obtained from 47.8 mg (0.1 mmol) of 10-*O*-propargylated ginkgolide C (**3″**). ^1^H-NMR (600 MHz, DMSO-*d_6_*) δ 8.83 (s, 1H), 7.88 (dt, *J* = 7.9, 1.1 Hz, 2H), 7.63 (dd, *J* = 8.8, 7.2 Hz, 3H), 7.53 (t, *J* = 7.4 Hz, 1H), 6.48 (s, 1H), 6.23 (s, 1H), 5.64 (t, *J* = 5.3 Hz, 2H), 5.46 (d, *J* = 12.2 Hz, 1H), 5.32 (s, 1H), 4.99 (d, *J* = 4.2 Hz, 2H), 4.63 (d, *J* = 7.0 Hz, 1H), 4.32 (s, 1H), 4.17 (dd, *J* = 7.0 Hz, 4.6 Hz, 1H), 2.98–2.78 (m, 1H), 1.99 (s, 1H), 1.57 (d, *J* = 12.5 Hz, 1H), 1.43–1.35 (m, 2H), 1.32–1.27 (m, 1H), 1.24 (s, 2H), 1.17 (t, *J* = 7.1 Hz, 1H), 1.13 (d, *J* = 7.1 Hz, 4H), 1.10 (s, 3H), 1.07 (s, 9H). ^13^C-NMR (150 MHz, DMSO-*d_6_*) δ 176.79, 172.94, 170.86, 162.28, 144.14, 136.89, 130.50, 122.14, 120.73, 110.04, 99.08, 93.14, 82.93, 75.47, 74.09, 67.05, 64.03, 63.52, 60.84, 49.35, 42.03, 35.12, 32.08, 19.09(3C), 14.32, 8.34. HRMS (ESI): *m*/*z* calcd for C_29_H_31_N_3_O_11_ [M + H] ^+^: 598.2039, found 598.2003.

*10*-*O-(1-(2-Methylphenyl)-1H-1,2,3-triazole) ginkgolide C* (**5″n**). Following the described procedure, 28.7 mg (47%) of compound **5″n** were obtained from 47.8 mg (0.1 mmol) of 10-*O*-propargylated ginkgolide C (**3″**). ^1^H-NMR (600 MHz, DMSO-*d_6_*) δ 8.47 (s, 1H), 7.50 (d, *J* = 2.2 Hz, 2H), 7.44 (d, *J* = 1.4 Hz, 2H), 6.48 (s, 1H), 6.23 (s, 1H), 5.68 (d, *J* = 4.7 Hz, 1H), 5.66 (dd, *J* = 6.2 Hz, 2.5 Hz, 1H), 5.49–5.43 (m, 1H), 5.32 (s, 1H), 5.02–5.00 (m, 2H), 5.00–4.96 (m, 1H), 4.65–4.61 (m, 1H), 4.17 (dd, *J* = 7.0 Hz, 4.7 Hz, 1H), 2.86 (d, *J* = 7.1 Hz, 1H), 2.16 (s, 3H), 1.13 (d, *J* = 7.1 Hz, 4H), 1.10 (d, *J* = 3.7 Hz, 6H), 1.08 (s, 9H). ^13^C-NMR (150 MHz, DMSO-*d_6_*) δ 176.81, 172.95, 170.89, 143.24, 136.51, 133.52, 131.93, 130.49, 127.57, 126.41, 125.27, 110.03, 99.05, 93.12, 82.92, 79.36, 75.41, 74.07, 69.35, 67.06, 64.05, 60.27, 49.38, 42.03, 32.10, 21.23(3C), 17.86, 8.34. HRMS (ESI): *m*/*z* calcd for C_30_H_33_N_3_O_11_ [M + H] ^+^: 612.2195, found 612.2157.

*10*-*O-(1-(2-Cyanophenyl) -1H-1,2,3-triazole) ginkgolide C* (**5″p**). Following the described procedure, 24.3 mg (39%) of compound **5″p** were obtained from 47.8 mg (0.1 mmol) of 10-*O*-propargylated ginkgolide C (**3″**). ^1^H-NMR (600 MHz, DMSO-*d_6_*) δ 8.78 (s, 1H), 8.17 (dd, *J* = 7.7, 1.4 Hz, 1H), 7.99 (td, *J* = 7.8 Hz, 1.5 Hz, 1H), 7.88 (dd, *J* = 8.2 Hz, 1.1 Hz, 1H), 7.79 (td, *J* = 7.7 Hz, 1.1 Hz, 1H), 6.48 (s, 1H), 6.23 (s, 1H), 5.64 (d, *J* = 6.1 Hz, 1H), 5.60 (d, *J* = 4.7 Hz, 1H), 5.49 (d, *J* = 12.4 Hz, 1H), 5.32 (s, 1H), 5.03 (d, *J* = 12.4 Hz, 1H), 5.01 (d, *J* = 4.2 Hz, 1H), 4.63 (d, *J* = 7.0 Hz, 1H), 4.17 (dd, *J* = 7.0 Hz, 4.7 Hz, 1H), 3.97 (ddd, *J* = 12.5 Hz, 6.1 Hz, 4.2 Hz, 1H), 2.86 (q, *J* = 7.1 Hz, 1H), 1.57 (d, *J* = 12.5 Hz, 1H), 1.13 (d, *J* = 7.1 Hz, 3H), 1.08 (s, 9H). ^13^C-NMR (150 MHz, DMSO-*d_6_*) δ 176.78, 172.90, 170.87, 143.98, 135.47, 126.10, 124.92, 116.26, 110.05, 107.36, 99.11, 93.18, 82.95, 79.34, 75.46, 74.18, 74.07, 67.04, 64.02, 63.40, 60.25, 49.37, 42.03, 32.10, 27.01, 22.55, 21.24(3C), 8.35. HRMS (ESI): *m*/*z* calcd for C_30_H_30_N_4_O_11_ [M + H] ^+^: 623.1991, found 623.1956.

*10-O-(1-Benzyl-1H-1,2,3-triazole) ginkgolide C* (**5″ff**). Following the described procedure, 26.9 mg (44%) of compound **5″ff** were obtained from 47.8 mg (0.1 mmol) of 10-*O*-propargylated ginkgolide C (**3″**). ^1^H-NMR (600 MHz, DMSO-*d_6_*) δ 8.19 (s, 1H), 7.40–7.37 (m, 2H), 7.35–7.32 (m, 3H), 6.46 (s, 1H), 6.20 (s, 1H), 5.73 (s, 1H), 5.62 (s, 3H), 5.35 (d, *J* = 12.2 Hz, 1H), 5.25 (s, 1H), 4.97 (d, *J* = 4.2 Hz, 1H), 4.87 (d, *J* = 12.2 Hz, 1H), 4.61 (d, *J* = 7.2 Hz, 1H), 4.13 (d, *J* = 5.7 Hz, 1H), 3.90 (d, *J* = 11.4 Hz, 1H), 2.84 (q, *J* = 7.1 Hz, 1H), 1.55 (dd, *J* = 12.5 Hz, 5.2 Hz, 1H), 1.17 (t, *J* = 7.1 Hz, 3H), 1.12 (d, *J* = 7.1 Hz, 4H), 1.10–1.09 (m, 2H), 1.03 (s, 9H). ^13^C-NMR (150 MHz, DMSO-*d_6_*) δ 176.81, 172.93, 170.87, 143.32, 136.15, 129.29(2C), 128.75, 128.56, 123.60, 110.01, 98.96, 93.02, 82.83, 79.33, 75.34, 74.01, 67.04, 64.00, 63.56, 60.27, 53.51, 49.30, 42.03, 32.04, 29.07, 21.23(3C), 8.27. HRMS (ESI): *m*/*z* calcd for C_30_H_33_N_4_O_11_ [M + H]^+^: 612.2195, found 612.2156.

*10-O-(((Anthracen-2-yloxy)methyl)-1H-1,2,3-triazole) ginkgolide C* (**5″gg**). Following the described procedure, 32.5 mg (51%) of compound **5″gg** were obtained from 47.8 mg (0.1 mmol) of 10-*O*-propargylated ginkgolide C (**3″**). ^1^H-NMR (600 MHz, DMSO-*d_6_*) δ 8.25 (s, 1H), 7.93 (dd, *J* = 7.7, 1.4 Hz, 1H), 7.74 (td, *J* = 7.7 Hz, 1.4 Hz, 1H), 7.58 (td, *J* = 7.7 Hz, 1.2 Hz, 1H), 7.40 (dd, *J* = 7.8 Hz, 1.1 Hz, 1H), 6.46 (s, 1H), 6.20 (s, 1H), 5.84 (s, 2H), 5.70 (d, *J* = 4.8 Hz, 1H), 5.62 (d, *J* = 6.1 Hz, 1H), 5.37 (d, *J* = 12.2 Hz, 1H), 5.26 (s, 1H), 4.98 (d, *J* = 4.2 Hz, 1H), 4.90 (d, *J* = 12.1 Hz, 1H), 4.61 (d, *J* = 7.0 Hz, 1H), 4.13 (dd, *J* = 7.1 Hz, 4.4 Hz, 1H), 3.90 (dt, *J* = 12.4 Hz, 4.7 Hz, 1H), 2.84 (q, *J* = 7.0 Hz, 1H), 2.50 (s, 3H), 1.55 (d, *J* = 12.5 Hz, 1H), 1.18 (t, *J* = 7.1 Hz, 3H), 1.12 (d, *J* = 7.2 Hz, 3H), 1.04 (s, 9H). ^13^C NMR (150 MHz, DMSO-*d_6_*) δ 176.83, 172.94, 170.92, 143.37, 138.90, 134.36, 133.88, 130.11, 129.85, 124.19, 117.47, 111.82, 110.02, 98.99, 82.84, 79.33, 75.38, 74.15, 74.01, 67.05, 64.01, 63.53, 60.27, 51.69, 49.33, 42.03, 32.04, 21.22 (3C), 8.28. HRMS (ESI): *m*/*z* calcd for C_31_H_32_N_4_O_11_ [M + H]^+^: 637.2148, found 637.2117

### 3.2. Antiplatelet Aggregation Activity Assay

The in vitro antiplatelet aggregation activity of the newly synthesized 10-substituted 1,2,3-triazole-ginkgolide derivatives was tested by the method of Born [26]. Blood samples (2 mL) from male New Zealand rabbits (2–2.5 kg body weight) were drawn into vacutainer tubes containing 200 μL of 3.2% sodium citrate. Platelet-rich plasma (PRP) was prepared by centrifuging the blood at 250× *g* for 10 min at 4 °C. The PRP was diluted with platelet-poor plasma obtained by further centrifuging at 3000× *g* for 10 min. The remaining blood was further centrifuged at 1600× *g* for 5 min to obtain platelet-poor plasma (PPP) as control group. Platelet aggregation was induced by PAF (10 nM) after incubating platelets with different concentrations of samples, and the maximum rate of platelet aggregation (RPA%) within 5 min was measured with a Helena Platelet aggregometer instrument [27]. The inhibition ratio was calculated according to the following formula:

Inhibition ratio (%) = (1−(RPA% of test group) / (RPA% of control group)) × 100%

In primary screening, the activities were expressed directly as inhibition ratio at 50 nM concentration. The activity of the most active ginkgolide-1,2,3-triazole derivatives was further expressed as the IC_50_ value (the concentration required to inhibit platelet aggregatory response by 50%). The values shown in the tables were calculated by linear regression from a single experimental curve with no less than four data points, each point being the mean of the percentage inhibition at a given concentration obtained from three independent experiments.

### 3.3. LDH Assay

#### 3.3.1. Preparation of H9c2 Cells

H9c2 cardio myoblast cells were grown in DMEM supplemented with 10% FBS, 100 U/mL penicillin-streptomycin. Cells were cultured at 37 °C with 5% CO_2_. The cells were subcultured when they reached 70–80% confluence [28]. Then the cells were seeded at 1 × 10^4^ cells per well in 96-well plate and incubated overnight. Then the cells were exposed to ginkgolide-1,2,3-triazole derivatives (1 μM and 10 μM).

#### 3.3.2. Preparation of Washed Platelets

Rat blood was collected in 3.8% sodium citrate vacuum anticoagulant tubes and centrifuged at 100× *g* for 15 min to obtain platelet-rich plasma (PRP). The PRP was centrifuged at 1000× *g* for 10 min at 37 °C. Then the platelet pellets were suspended in Tyrode’s solution (pH 7.4). The washed platelets were adjusted to 3.6 × 10^8^ platelets/mL. Washed platelets (3.6 × 10^8^ cells/mL) were pre-incubated with ginkgolide-1,2,3-triazole derivatives (1 μM and 10 μM) or 0.1% DMSO for 20 min at 37 °C, then centrifuged at 1700× *g*, 10 min and the supernatant collected [29].

#### 3.3.3. Measurement of Lactate Dehydrogenase (LDH)

The assays to measure of LDH release were conducted in 96-well plates according to the manufacturer’s protocol. The LDH levels were measured at 490 nm using a microplate reader (Thermo Fisher Scientific, Waltham, MA, USA). Cell cytotoxicity was also detected by the LDH activity assay kit. Cells incubated with 0.1% dimethyl sulfoxide (DMSO) served as the control group [28,29].

## 4. Conclusions

In summary, a series of ginkgolide-1,2,3-triazole conjugates were synthesized through a copper(I)-catalyzed Huisgen 1,3-dipolar cycloaddition reaction of the corresponding 10-*O*-propargylated ginkgolides with benzyl, phenyl and heterocyclic azides. Five of them (compounds **5a**, **5p**, **5ff**, **5gg** and **5′a**) displayed promising antiplatelet aggregation activities with IC_50_ values ranging from 5–21 nM. Compounds **5ff** and **5gg**, having a benzyl group attached at the triazole nucleus were the best among the series of compounds. The most active compounds may be regarded as safe towards normal cells and platelets at therapeutic concentrations.

## Figures and Tables

**Table 1:** Synthesized azides **4a**–**4ee**.

Entry	Azide	Ar	Yield
1	**4a**	Ph	77
2	**4b**	2-Cl-Ph	88
3	**4c**	3-Cl-Ph	91
4	**4d**	4-Cl-Ph	75
5	**4e**	2-Cl, 6Cl-Ph	72
6	**4f**	2-Cl, 4Cl-Ph	71
7	**4g**	3-Cl, 4Cl-Ph	87
8	**4h**	3-F-Ph	88
9	**4i**	3-CF_3_, 4-Cl-Ph	62
10	**4j**	4-OCF_3_-Ph	86
11	**4k**	3-CF_3_, 5-CF_3_Ph	91
12	**4l**	4-tertbutyl-Ph	76
13	**4m**	3-Br-Ph	62
14	**4n**	2-Me-Ph	75
15	**4o**	2-OH-Ph	92
16	**4p**	2-CN-Ph	88
17	**4q**	3-Me-Ph	74
18	**4r**	3-isopropyl-Ph	87
19	**4s**	3-COOEt-Ph	78
20	**4t**	3-COOH-Ph	78
21	**4u**	4-Me-Ph	65
22	**4v**	4-OMe-Ph	87
23	**4w**	4-F-Ph	74
24	**4x**	4-Br-Ph	63
25	**4y**	4-CF_3_-Ph	77
26	**4z**	4-NO_2_-Ph	67
27	**4aa**	4-COOMe-Ph	87
28	**4bb**	4-COOH-Ph	76
29	**4cc**	3-OMe,5-OMe-Ph	79
30	**4dd**	3-Cl, 4F-Ph	87
31	**4ee**	3-pyridine	68

**Table 2:** Synthesized azides **4ff**–**4gg.**

Entry	Azide	Ar	Yield
1	**4ff**	Bn	82
2	**4gg**	2-Me-Bn	77

**Table 3:** In vitro antiplatelet aggregation activity study of some of ginkgolide-1,2,3-triazole derivatives.

Compound	PAF-Induced Platelet Aggregation IC_50_ (nM)	Compound	PAF-Induced Platelet Aggregation IC_50_ (nM)
**1**	130.1 ± 20.0	**5ff**	5.31 ± 1.84
**1′**	580.4 ± 50.3	**5gg**	5.68 ± 0.71
**1″**	7540 ± 662	**5′a**	18.55 ± 2.25
**5a**	7.74 ± 1.89	**5′ff**	25.37 ± 4.25
**5n**	20.63 ± 2.84	**5′gg**	71.47 ± 6.26
**5p**	10.19 ± 2.03	**5″gg**	37.68 ± 4.17
**5q**	44.33 ± 4.42		
